# Cooperative Effects in the Inverse Coordination Complexes of Aromatic Azines and Tin(IV) Halides

**DOI:** 10.1021/acs.jpca.6c03412

**Published:** 2026-07-05

**Authors:** Piotr Matczak

**Affiliations:** Department of Physical Chemistry, Faculty of Chemistry, University of Lodz, Pomorska 163/165, 90236 Lodz, Poland

## Abstract

In this work, a series of model inverse coordination complexes composed of one aromatic azine molecule (pyrimidine, pyrazine, 1,2,4-triazine, 1,3,5-triazine, and 1,2,4,5-tetrazine) and two SnX_4_ (X = F, Cl, Br, I) molecules has been investigated using quantum chemical calculations. These 1:2 complexes demonstrate a rare structural motif—as evidenced by the Cambridge Structural Database (CSD)—in which a single “naked” azine center forms two N→Sn coordinate bonds simultaneously. Calculations reveal that the two bonds influence one another and, consequently, the 1:2 complexes exhibit longer N→Sn bonds and weaker interactions between the azine and SnX_4_ fragments than the corresponding 1:1 conventional complexes. Thus, the studied inverse coordination complexes are characterized by negative cooperative effects (or anticooperativity) between their N→Sn bonds. This conclusion is supported by an occurrence of the destabilizing three-body nonadditive contribution to the total interaction energy calculated at the CCSD­(T)/CBS level of theory. The origin of the anticooperativity has been unveiled by the analysis of electron charge distribution and the fundamental physical nature of individual many-body contributions to the total interaction energy. Overall, this work shows how cooperative effects, together with the proper choice of azine center, can modulate the strength of N→Sn bonds in tin­(IV) inverse coordination complexes.

## Graphical abstract

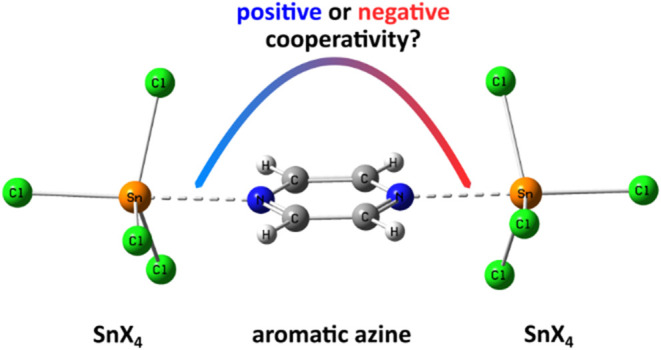

## Introduction

Since the early years of the 20th century, halide complexes of tetravalent tin achieving a higher coordination number have been arousing considerable interest for a number of reasons.^1–5^ First, such complexes have found a wide range of synthetic,^6,7^ biological,^8,9^ and technological^10,11^ applications. Presently, their application in the synthesis of tin-based halide perovskites becomes particularly relevant to the area of energy production and storage.^12–14^ Another focus of attention is the diversity of coordination geometries around the metal center in tin­(IV) halide complexes and their related complex anions. A variety of geometries, that in general can be encountered around highly coordinated Sn­(IV) centers,^15^ allowed tin­(IV) halide complexes to be successfully employed in the construction of crystalline functionalized complexes and supramolecular networks.^9,16^ Therefore, over the past 30 years, the coordination chemistry of neutral and anionic tin­(IV) halide complexes has been reviewed on numerous occasions^17–20^ and this topic is still an area of intense activity.^21^

In addition to halogens, other ligands can also be coordinated to the metal in tin­(IV) halide complexes. In the field of coordination chemistry, monocyclic aromatic azines possessing at least two nitrogen atoms in the six-membered ring (named azines from now on) are a well-known class of N-heterocyclic ligands capable of binding a metal center in a simple σ-donor mode via the electron lone pair of their nitrogen.^22^ As a matter of fact, the abundance of such σ-donor sites in the ring of azines offers an opportunity for simultaneous coordination at more than one of these sites. In particular, separate metal atoms may be bound at the same time to different N atom sites in the ring of azines. In that regard, the role of azines is usually described as a bridging ligand between two or more metal atoms.^23^ The simple and controllable coordination mode and small steric hindrance of azines^24,25^ provoked the synthesis of polynuclear coordination networks from the building blocks of azines coordinated to the centers of various metals,^23^ including tin.^26–28^ A new look at the coordination chemistry of crystalline polynuclear tin­(IV) halide complexes with azines can be provided by the concept of inverse coordination.^29^ This novel concept was introduced to distinguish coordination complexes formed around a nonmetal donor center surrounded by a few metallic acceptors with their own terminal ligands to satisfy the coordination numbers of these metals. In other words, the arrangement of Lewis acidic and basic sites in inverse coordination complexes is opposite to that occurring in conventional coordination complexes. In the spirit of this concept, a single azine molecule can serve as an inverse coordination center for two or more Sn­(IV) atoms bearing their own terminal ligands – halogens, for example.^30^

The bonding interaction between the Lewis acidic site of metal and the Lewis basic site at the N atom of a nitrogen-containing ligand in tin­(IV) complexes is frequently classified as the coordinate (or donor–acceptor) bond of N→Sn type.^31–35^ For longer distances between interacting N and Sn atoms, their bonds have however been termed the tetrel ones^19^ and these constitute a new type of noncovalent bonds.^36^ A simple geometrical criterion was proposed to differentiate between the coordinate and tetrel types of nitrogen–tin bonds in crystalline complexes.^19^ More recently, the distinction between these types can also be established using a quantum chemical analysis of the total static and electrostatic potentials along bond lines.^37^

The coexistence of a few nonmetal–metal coordinate bonds involving the same inverse coordination center gives rise to possible cooperative effects, coming out of how these bonds influence each other.^38^ These effects may not only change the strengths of the coordinate bonds but also contribute to the stability and properties of the whole complex. When coordinate bonds strengthen each other, the corresponding effect is termed as positive cooperativity. Conversely, negative cooperativity (or anticooperativity) refers to a mutual weakening in the strengths of coordinate bonds. A wealth of information on the cooperative effects of metal–ligand bonds for conventional coordination complexes is available from literature.^39,40^ A nonexhaustive list of examples of conventional complexes presenting metal–ligand cooperativity also includes those with Sn­(IV) centers.^41^ As for the complexes of tin­(IV) halides and aromatic azines, the strengthening of N→Sn coordinate bonds upon introducing another pyridine ligand into the 1:1 complexes of SnX_4_ (X = F, Cl, Br, I) and pyridine was established using a computational quantum chemical approach.^42^ By contrast, no studies have so far been dedicated to cooperative effects in tin­(IV) inverse coordination complexes and, generally, little is still known about such effects in polynuclear complexes.^38^ The present work aims at providing part of the missing knowledge on the nature and origin of cooperative effects between N→Sn bonds in azine-centered ditin­(IV) inverse coordination complexes. To that end, a series of model 1:2 complexes formed by a single “naked” (that is, nonfunctionalized) azine molecule **1**–**5** and two SnX_4_ molecules **A**–**D** (Figure 1) has been investigated using computational quantum-chemical methods.

**Figure 1:**
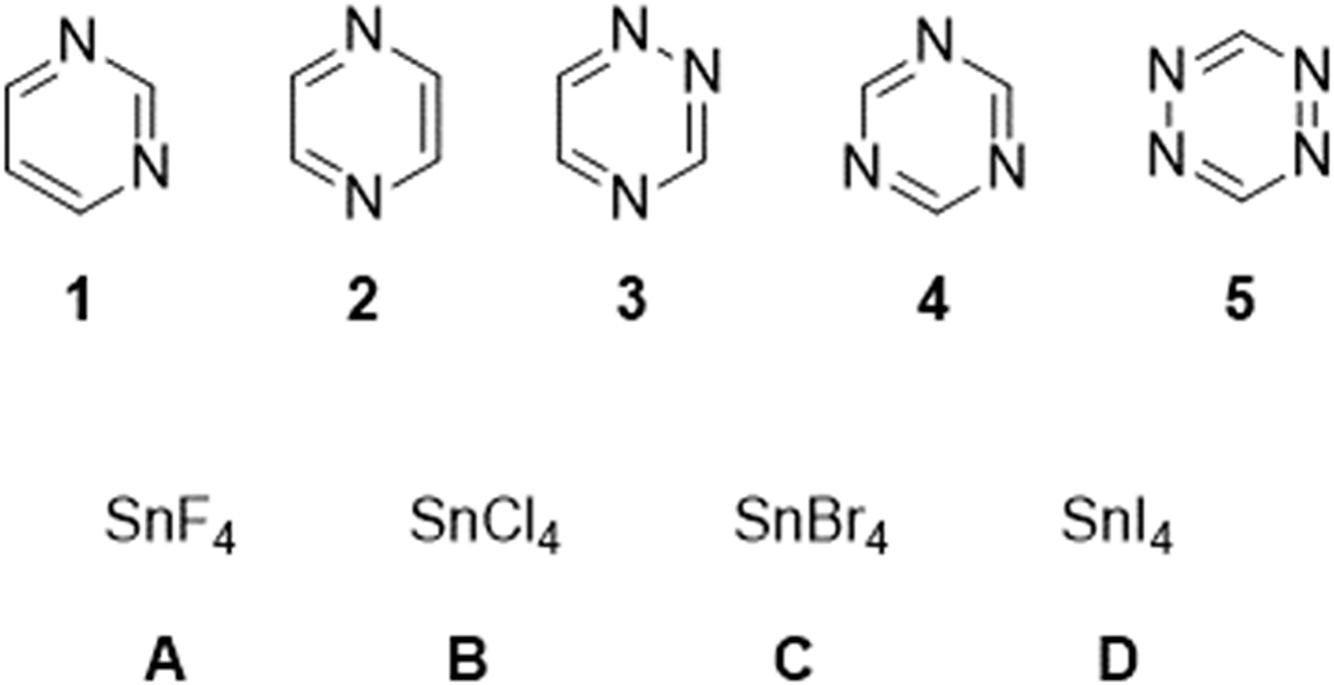
Azines **1**–**5** and tin­(IV) halides **A**–**D** considered in this work.

## Methods

Geometry optimizations and harmonic vibrational frequency calculations for the isolated molecules of **1**–**5**, **A**–**D**, and their 1:2 complexes (denoted further as **1A**_2_, **1B**_2_ and so on) were carried out at a dispersion-corrected DFT level of theory. The BP-D functional^43–45^ was combined with the aug-cc-pVTZ orbital basis set^46^ and its pseudopotential-based “PP” extension^47,48^ assigned to the atoms of Sn, Br, and, I – the resulting basis set is abbreviated here as aTZ. The optimized geometries were verified to be local energy minima without any imaginary frequencies. It was proved in previous studies that the BP-D/aTZ level of theory is capable of providing reliable geometrical structures of complexes with N→Sn coordinate bonds.^49–51^ At this level, each complex was characterized by its complexation energy (*E*_complex_) and the total interaction energy (*E*_int_). The *E*_int_ energy was also calculated using the modern dispersion-corrected r^2^SCAN-D density functional^52–54^ in combination with aTZ, and the CCSD­(T) method,^55–57^ together with an extrapolation to the complete basis set (CBS) limit.^58–61^ The basis set superposition error in *E*_int_ was eliminated by the counterpoise method.^62^ The *E*_int_ energy was decomposed into many-body contributions, as well as into fundamental physical forces within the framework of the localized molecular orbital energy decomposition analysis (LMOEDA).^63^ Supplementary insights into the nature of interactions were provided by the quantum theory of atoms in molecules (QTAIM)^64^ and the natural bond orbital (NBO) method,^65^ including its natural population analysis (NPA).^66^

Geometry optimizations and the subsequent calculations of harmonic vibrational frequencies, complexation and interaction energies, and LMOEDA components were carried out using the TURBOMOLE 7.7 program.^67^ Extended wave function files (WFX) were provided by the Gaussian 09 D.01 program.^68^ The QTAIM and NBO analyses were done with the AIMAll 19.10.12 ^69^ and NBO 6.0 ^70^ programs, respectively.

Further details regarding the aforementioned computational methodology and its validation can be found in Sections S1 and S2 in the Supporting Information.

## Results and Discussion

### CSD Survey

First, to place the complexes theoretically studied here into experimental perspective, the Cambridge Structural Database (CSD version 6.0 with updates up to April 2025)^71^ was searched for crystal structures featuring both functionalized and “naked” azines **1**–**5** as inverse coordination centers for Sn­(IV). The search revealed a small number of crystal structures with **1**, **2** or **4** acting as inverse coordination centers for two Sn­(IV) acceptors (14 CSD entries in total; Table S11 in the Supporting Information), while the CSD lacks any entries for ditin­(IV) inverse coordination complexes with **3** and **5**. In part of these structures, the Sn­(IV) atoms are also connected with at least one halogen as their terminal ligand. To be precise, six inverse coordination complexes (CSD codes: CORMIF,^28,72^ FARVID,^27,28^ FIHMOY,^26^ FIHMUE,^26^ YELLUX,^34^ YELMIM^34^) were found to possess one or two terminal Cl ligands of Sn­(IV) while one crystal structure (FARVOJ^27^) discloses two terminal Br ligands attached to Sn­(IV). The coordination geometry about Sn­(IV) in the crystal structures of dichlorodiorganostannanes with nonfunctionalized **2** (CORMIF and FARVID) conforms quite well to trigonal bipyramidal, with the Cl and N atoms in the axial positions. Overall, the CSD survey provides experimental evidence for the ability of azines to play the role of an inverse coordination center for two halotin­(IV) acceptors yet the resulting structural motif remains a rare phenomenon in crystalline solids, as evidenced by the small number of corresponding entries in the CSD.

### Structure and Stability

In the inverse coordination complexes under study, the molecule of **1**–**5** attaches two molecules of **A**–**D**, leading to the formation of two N→Sn coordinate bonds. According to the Lewis acid–base theory, the σ-donor sites at two N atoms of the inverse coordination center interact with separate acceptor Sn atoms. Two Sn­(IV) acceptors are coordinated to the N-donors at positions 1 and 3 in the ring of **1** and **4**, and at positions 1 and 4 in the ring of **2** (Figure 2). The N atoms of **3** and **5** afford both 1,3- and 1,4-coordination with two Sn atoms of **A**–**D**.

**Figure 2:**
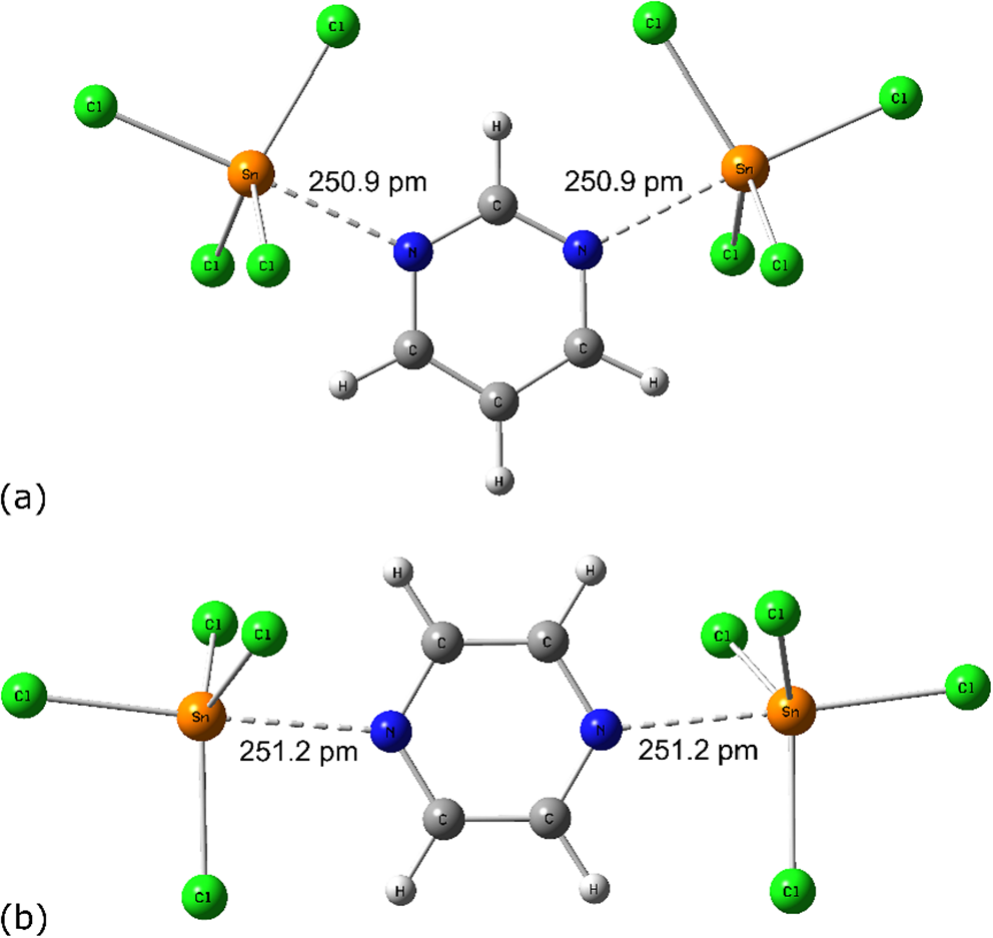
Optimized lowest-energy geometrical structures of complexes (a) **1B**_2_ and (b) **2B**_2_. Their N→Sn bond lengths are also shown.

The optimized geometries of the complexes show that the donor atom of the azine center and the terminal halogen ligands around the Sn atoms are arranged in a distorted trigonal bipyramidal geometry with the N atom located at the axial position (Figure 2), which is in line with the immediate geometry around Sn­(IV) in the crystal structures of dichlorodiorganostannane complexes with **2** acting as an inverse coordination center.^27,28^ Moreover, such an arrangement is well-known for pentacoordinate Sn­(IV) atoms in their 1:1 complexes with neutral N-donor ligands, including electron donor solvents.^31^ The angle of each N→Sn–X axis in the geometry-optimized complexes deviates by no more than 5° from the ideal value of 180°. Table 1 presents the N→Sn bond lengths (*d*) calculated for the complexes of **1** and **2**, exemplifying the 1,3- and 1,4-coordination of the azine center, respectively (results for the remaining complexes are listed in Table S12). The *d* values of these diazine complexes fall into a range from 234.5 to 271.0 pm, which is typical of intermolecular N→Sn coordinate bonds in hypervalent Sn­(IV) complexes.^31^ It should however be noted that, according to the geometrical criterion based on the sum of covalent radii,^19^ the *d* values from the upper end of this range may actually be attributed to the tetrel bond (or a hybrid one) rather than the coordinate bond. The *d* value of **2B**_2_ is shorter than the N→Sn bond lengths observed for two inverse coordination complexes of dichlorodiorganostannanes with **2** (268.3 and 274.5 pm).^28^ This is in agreement with the reduced Lewis acidity of the Sn­(IV) atom bonded with two organic groups, as compared to that of **B**.

**Table 1:** Geometrical (in pm) and Energetic Parameters (in kJ mol^–1^) for the Complexes of two SnX_4_ Molecules Coordinated to Pyrimidine or Pyrazine

				*E*_def_		
complex	*d*	*E*_complex_	*E*_in_ ^a^	azine	2SnX_4_	ΔZPVE
**1A**_2_	234.5	–191.2	–268.2 (−266.6)	6.6	60.3	10.0
**1B**_2_	250.9	–98.6	–177.1 (−176.4)	3.9	66.1	8.5
**1C**_2_	258.5	–87.3	–153.5 (−153.9)	2.7	56.7	6.9
**1D**_2_	271.0	–79.3	–131.4 (−127.7)	1.8	44.7	5.7
**2A**_2_	235.0	–190.6	–265.0 (−263.5)	4.2	59.6	10.7
**2B**_2_	251.2	–97.0	–173.6 (−172.8)	2.3	65.7	8.5
**2C**_2_	257.9	–83.7	–152.4 (−152.7)	1.9	59.5	7.3
**2D**_2_	269.1	–71.4	–127.5 (−123.2)	1.4	48.7	5.9

a Interaction energies including both scalar and spin–orbit relativistic corrections are given in parentheses.

Table 1 also presents the complexation energy (*E*_complex_) expressing the energetic effect associated with the formation of **1A**_2_–**1D**_2_ and **2A**_2_–**2D**_2_. The negative values of *E*_complex_ prove that the formation of these complexes from the isolated molecules is energetically favorable. The *E*_complex_ energy is determined mostly by the stabilizing contribution from the total interaction between three molecular fragments in each complex (*E*_int_), while the smaller contributions from the structural deformations of molecules (*E*_def_) and the change in zero-point vibrational energies (ΔZPVE) are positive and thus destabilizing. The value of *E*_def_ for two SnX_4_ molecules is 1 order of magnitude larger than the *E*_def_ energy of azine. This is due to the fact that the tetrahedral geometry around the Sn atoms in the isolated SnX_4_ molecules undergoes a significant change upon complexation as well as the Sn–X bonds elongate.

The *E*_int_ contribution to *E*_complex_ takes account of the scalar relativistic correction obtained from small-core relativistic pseudopotentials for heavier elements. The incorporation of another relativistic correction, which is due to the splitting of inner atomic shells by spin–orbit coupling, into *E*_int_ weakens the total interaction in the studied complexes to a small degree (Tables 1 and S12). Unsurprisingly, the effect of the spin–orbit relativistic correction on *E*_int_ is largest for the complexes containing SnI_4_ for which the strength of their total interaction is decreased by 4.3 kJ mol^–1^ at most (for **2D**_2_).

Complexes **1A**_2_–**1D**_2_ and **2A**_2_–**2D**_2_ are representative to make a comparison between the 1,3- and 1,4-coordination of the diazine center. The values of *E*_complex_ indicate that **1A**_2_–**1D**_2_ gain an extra energetic stabilization in comparison to **2A**_2_–**2D**_2_. The preference of 1,3-coordination arises from the greater donor ability of N atoms in **1** than in **2**. This greater donor ability is reflected in the increased nucleophilicity index condensed on the N atoms of **1** (Section S3) and it results in stronger interactions with the electrophilic site of SnX_4_, leading to the more stabilizing *E*_int_ energies of **1A**_2_–**1D**_2_. However, this is not conclusive for all complexes containing the tri- and tetrazine centers. The *E*_complex_ energies of **3A**_2_ and **5A**_2_ reveal the preference of 1,4-coordination (Table S12).

Another trend that can be deduced from Table 1 is the effect of terminal halogen ligands on the structure and stability of the series of complexes with a fixed diazine center. The *d* values systematically grow as the X ligands become heavier and heavier in each series. The growing N→Sn bond length is accompanied by a decreased stability and weaker interactions, as evidenced by less negative values of *E*_complex_ and *E*_int_, respectively. The halogen dependence of these quantities is also valid for the series of the remaining complexes containing the tri- and tetrazine centers (Table S12). Moreover, such a halogen dependence was previously reported in many theoretical studies of conventional coordination complexes of SnX_4_ with various N-donor ligands^42,73,74^ and it was essentially in agreement with available experimental results.^75–78^ In the case of inverse coordination complexes, the manifestation of halogen dependence in growing N–metal bond lengths was reported for the crystal structures of dirhenium­(IV) complexes with **2**.^79^ The effect of terminal halogen ligands can be understood in terms of Lewis acid–base theory because tin­(IV) halides are good Lewis acids. Their Lewis acidity decreases in the sequence **A** > **B** > **C** > **D** and this sequence is confirmed by decreasing local electrophilicity on their Sn atoms (Table S2). The region of electron deficiency at the extension of each Sn–X bond in SnX_4_ is also manifested in the positive values of the surface electrostatic potential, and its maximal values strictly follow the aforementioned sequence (Table S2).

Aside from the aforementioned effect of SnX_4_, the azine center may also produce certain effects on the geometrical and energetic features of the complexes under study. Table 2 lists the results calculated for a series of complexes containing the same kind of tin­(IV) halide in order to dissect the effects associated with the number of N atoms and their positioning in the azine ring. The growing number of N atoms in the azine ring results in a gradual elongation of N→Sn bonds and, simultaneously, in the reduced stability of complexes due to the smaller strength of the total interaction between the molecular fragments in the complexes. The effect of the number of N atoms relies on the decreasing nucleophilicity of N atoms in the sequence **1** > **4** > **5**, also manifested in the minimal values of the surface electrostatic potential in the regions occupied by the electron lone pairs belonging to the N-donors of these azines (Table S3). The same dependence on the number of N atoms was previously observed for the geometrical and energetic parameters calculated for conventional coordination complexes of azines with stannylenes,^80^ as well as with ions of other metals^81^ and CO_2_.^82^ As for inverse coordination complexes with “naked” azine centers, an elongation of N–Re bonds was observed in the crystal structures of dirhenium­(IV) bromide complexes after replacing **1** with **4** as the inverse coordination center.^79^

**Table 2:** Geometrical (in pm) and Energetic Parameters (in kJ mol^–1^) for the Complexes Formed by the 1,3-Coordination of Two SnCl_4_ Molecules to an Azine Center

				*E*_def_		
complex	*d*	*E*_complex_	*E*_int_ ^a^	azine	2SnX_4_	ΔZPVE
**1B**_2_	250.9	–98.6	–177.1 (−176.4)	3.9	66.1	8.5
**3B**_2_	255.1; 258.1^b^	–79.2	–142.9 (−142.4)	3.7	52.3	7.8
**4B**_2_	255.4	–80.1	–144.9 (−144.5)	3.6	54.0	7.2
**5B**_2_	265.1	–58.9	–108.4 (−108.0)	3.6	38.8	7.1

a Interaction energies including both scalar and spin–orbit relativistic corrections are given in parentheses.

b Results for N→Sn involving the N atoms at positions 2 and 4, respectively.

Complexes **3B**_2_ and **4B**_2_ from Table 2 illustrate how the positioning of N atoms in the ring of triazines affects the 1,3-coordination with 2SnCl_4_. The nonsymmetrical triazine **3** forms a less stable complex with one N→Sn bond being slightly longer than the other one. The elongated bond involves the N atom at position 4 in the ring of **3** because this N atom shows a reduced electron donor ability, as compared to the N atoms at positions 1 and 2 (Table S3). In consequence, a weaker interaction with the electrophilic site of **B** is expected for N at position 4 than at position 2 in **3B**_2_. In general, the N atoms of **3** exhibit greater electron donor ability than the N atoms of **4** (Table S3) but the stability of **3B**_2_ is lower than that of **4B**_2_. This highlights the occurrence of contributions from other interactions than only the N→Sn ones to the overall stability of these complexes. Interestingly, the *d* values of **3D**_2_ and **4D**_2_ turn out to be large enough to reduce any secondary interactions and, therefore, **3D**_2_ shows a more negative *E*_int_ value than that of **4D**_2_ (Table S12), which is in accord with higher electron donor abilities for the N atoms of **3**.

### Interaction Energy Decomposition

Next, the *E*_int_ energy calculated at the CCSD­(T)/CBS level of theory for the studied inverse coordination complexes was partitioned into pairwise and nonadditive contributions (ε_int_) to estimate the importance of individual interactions between the molecular fragments in the stabilization of the complexes. The many-body analysis of *E*_int_ for the complexes containing the diazine center is presented in Table 3. A single ε_int_(azine,SnX_4_) contribution is listed in this table because both interactions between the azine center and each SnX_4_ fragment were identical in these complexes due to their symmetry. Unsurprisingly, both ε_int_(azine,SnX_4_) contributions are always stabilizing and they play the leading role in determining the magnitude of *E*_int_. The values of ε_int_(azine,SnX_4_) vary in a regular way that mimics the aforementioned trends in *E*_int_, such as the halogen dependence and the effects associated with the number of N atoms and their positioning in the azine ring (Tables 4 and S13). The contribution from the interaction energy between two SnX_4_ fragments is only marginal and its destabilizing role changes into the more and more stabilizing one with the growing atomic number of X. The destabilizing effect of ε_int_(SnX_4_,SnX_4_^′^) in the complexes containing two molecules of **A** has its origin in the repulsion between the fluorine terminal ligands bearing the significant negative charge due to the high ionicity of Sn–F bonds. For heavier halogen terminal ligands, the ε_int_(SnX_4_,SnX_4_^′^) contribution is a manifestation of weak van der Waals interactions between the SnX_4_ fragments. Such interactions were previously observed for the dimers of tetrahedral TiCl_4_ and TiBr_4_ molecules but not for TiF_4_.^83^ Interestingly, in the case of the 1,4-coordination, the longer distance between the SnI_4_ fragments of **2D**_2_ is favored by the dispersion interaction in comparison with the SnBr_4_ fragments arranged closer to one another in **2C**_2_.

**Table 3:** Many-Body Analysis of the Total Interaction Energy (in kJ mol^–1^) Calculated Using CCSD­(T)/CBS for the Complexes of Two SnX_4_ Molecules Coordinated to Pyrimidine or Pyrazine

complex	*E*_int_	ε_int_(azine,SnX_4_)^a^	ε_int_(SnX_4_,SnX_4_^′^)	ε_int_(azine,SnX_4_,SnX_4_^′^)
**1A**_2_	–295.1	–160.8	5.0	21.5
**1B**_2_	–191.8	–104.3	–1.3	18.2
**1C**_2_	–155.5	–81.6	–9.2	16.8
**1D**_2_	–120.2	–60.2	–8.2	8.3
**2A**_2_	–288.2	–158.3	3.4	25.0
**2B**_2_	–188.4	–101.6	0.5	14.4
**2C**_2_	–152.3	–80.7	–0.1	9.1
**2D**_2_	–118.5	–61.3	–1.7	5.9

a ε_int_(azine,SnX_4_) = ε_int_(azine,SnX_4_^′^)

**Table 4:** Many-Body Analysis of the Total Interaction Energy (in kJ mol^–1^) Calculated Using CCSD­(T)/CBS for the Complexes Formed by the 1,3-Coordination of Two SnCl_4_ Molecules to an Azine Center

complex	*E*_int_	ε_int_(azine,SnX_4_)^a^	ε_int_(SnX_4_,SnX_4_^′^)	ε_int_(azine,SnX_4_,SnX_4_^′^)
**1B**_2_	–191.8	–104.3	–1.3	18.2
**3B**_2_	–153.6	–88.1; −77.3^b^	–1.5	13.2
**4B**_2_	–155.0	–83.2	–1.4	13.0
**5B**_2_	–116.8	–63.5	–1.6	11.8

a ε_int_(azine,SnX_4_) = ε_int_(azine,SnX_4_^′^) unless two different values are given.

b Results for SnCl_4_ coordinated to the N atoms at positions 2 and 4, respectively.

The three-body nonadditive contribution to *E*_int_ adopts positive values for all the studied inverse coordination complexes (Tables 3 and S13). This indicates that a destabilizing nonadditive effect can be attributed to the interaction between three molecular fragments in these complexes. However, the value of ε_int_(azine,SnX_4_,SnX_4_^′^) for the 1,4-coordination in **5D**_2_ amounts to merely 0.4 kJ mol^–1^ and in this case the pairwise interactions might be termed practically additive, mostly because of their small strength. The values of *ε*_int_(azine,SnX_4_,SnX_4_^′^) are generally one or even 2 orders of magnitude smaller than the absolute values of *E*_int_. To be precise, the magnitude of ε_int_(azine,SnX_4_,SnX_4_^′^) does not exceed 12% of the absolute value of *E*_int_. In each series of complexes with a fixed azine center, the percentage share of this contribution is smallest for the complex containing two molecules of **D**. Positive values of three-body contribution have so far been reported for conventional 2:1 complexes of pyridine with tin­(IV) halides^42^ and for tin­(II) halides complexed to pyridine via mixed σ- and π-type binding modes.^51^

### Cooperative Effects

Finally, the cooperative effects between the N→Sn bonds in the studied inverse coordination complexes were quantified by comparing these 1:2 complexes with their 1:1 conventional coordination counterparts (denoted further as **1A**, **1B** and so on). As the focus in this work is on the inverse coordination complexes, the 1:1 complexes are not described here in greater detail – suffice it to say that their structure and stability essentially follow the trends discussed above for the 1:2 complexes (Section S4).

Figure 3 compares the *d* values calculated for both 1:1 and 1:2 complexes of **1** with tin­(IV) halides. From this figure it can be deduced that the introduction of another SnX_4_ molecule into a 1:1 complex elongates the N→Sn bond of the already coordinated SnX_4_ molecule. Generally, both N→Sn bonds of the 1:2 complexes with the symmetrical azine center are identical in length and simultaneously longer than the N→Sn bond in the corresponding 1:1 complexes (Table S14). The resulting N→Sn elongation in these 1:2 complexes ranges from 4.0 to 26.2 pm. In the series of the complexes with a fixed azine center, this elongation increases as the atomic number of halogens being the terminal ligands grows. As for the complexes containing the nonsymmetrical center **3**, the N→Sn bond involving the N-donor at position 4 usually gets longer more than the N→Sn bonds with the N-donors at positions 1 or 2. The coordinate bond from the N atom at position 4 in **3D**_2_ is elongated by as much as 46.5 pm, while the second N→Sn bond in this complex remains practically unchanged relative to that of **3D**. The elongation of N→Sn bonds in the 1:2 complexes is accompanied by lower values of *E*_def_ per SnX_4_ molecule than in the corresponding 1:1 complexes (Figure S4). It means that the tetrahedral molecular geometry of **A**–**D** undergoes smaller distortion in the 1:2 complexes.

**Figure 3:**
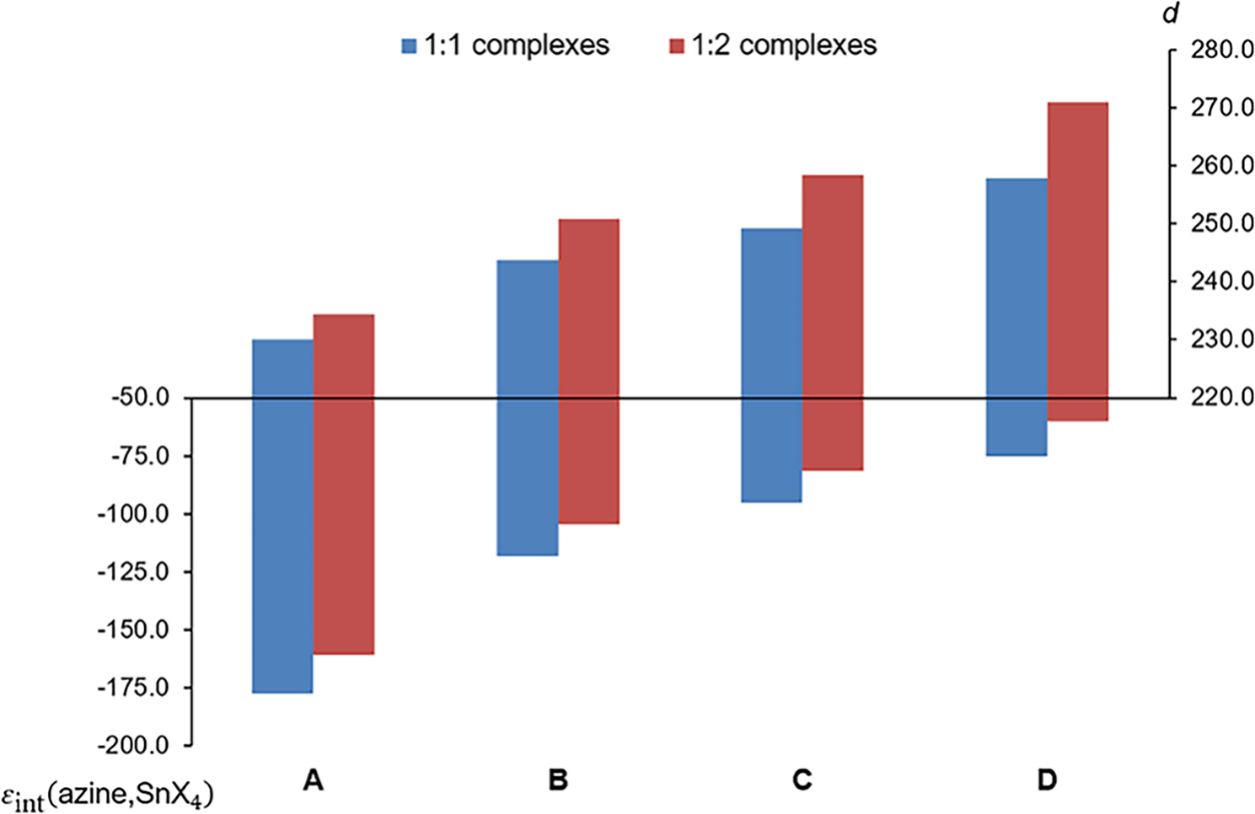
Variations in the N→Sn bond length (*d*, in pm) and the pairwise azine-SnX_4_ interaction (ε_int_(azine,SnX_4_), in kJ mol^–1^) among the 1:1 and 1:2 complexes of **1** and **A**–**D**.

To our knowledge, there has so far been no direct experimental evidence for the elongation of N→Sn bonds in the crystal structures of tin­(IV) halide complexes with “naked” azines as inverse coordination centers. There were, however, reported crystal structures of transition metal complexes involving “naked” azines in the roles of regular ligand and inverse coordination center.^79,84–86^ Comparison of N–metal bond lengths in mono- and dinuclear complexes of such transition metals as ruthenium^84,85^ or rhenium^79,86^ with **2** reveals that these bonds elongate by as much as several pm in the dinuclear complexes. In that light, the behavior of N→Sn bonds in the complexes studied here does not seem to be a rare exception in the coordination chemistry of inverse coordination complexes. The calculated magnitude of N→Sn elongation is however slightly larger than that deduced from the N–Re and N–Ru bond lengths in crystals, probably due to the neglect of crystal packing effects in the calculations.

The aforementioned elongation of N→Sn bonds is associated with a certain change in their nature. According to the QTAIM and NBO characteristics, the N→Sn bonds in the studied inverse coordination complexes are largely ionic with a certain covalent contribution that decreases as the atomic number of halogen terminal ligands grows (Section S5). The N→Sn bonds of the 1:2 complexes become less covalent in character upon attaching the second SnX_4_ molecule. This is demonstrated by the decreased values of such descriptors of the degree of covalency as the delocalization index^87^ and the Wiberg bond index^88^ for N→Sn, as well as the QTAIM parameters at the critical point on the bond path linking the atoms of N→Sn in the 1:2 complexes (Figure 4). The elongation of N→Sn bonds, along with the decrease in their partially covalent character, affect the strength of these bonds in the 1:2 complexes. Comparing each pairwise azine-SnX_4_ interaction in **1A**_2_–**1D**_2_ with the interaction between the molecular fragments in **1A**–**1D** reveals that the introduction of another SnX_4_ molecule into **1A**–**1D** weakens this interaction in the resulting 1:2 complexes (Figure 3, Table S14). The strength of this interaction in **1A**_2_–**1D**_2_ is reduced by 9.8 to 16.7 kJ mol^–1^. For the 1:2 complexes with the symmetrical azine center, the greatest reductions are always observed for X = F while heavier halogens in 2SnX_4_ usually produce a gradually diminishing effect on pairwise azine-SnX_4_ interactions. The coordination of SnX_4_ by the N-donor at position 4 in the nonsymmetrical azine center **3** is weaker even by 32.6 kJ mol^–1^, given that a prior coordination of SnX_4_ at position 1 or 2 has occurred. For **3D**_2_ the significant weakening of its pairwise azine-SnX_4_ interaction involving the N-donor at position 4 is associated with the appreciable elongation of the corresponding N→Sn bond. Thus, the negative cooperativity occurs between the N→Sn bonds in the studied inverse coordination complexes. The same phenomenon was observed between two tetrel bonds formed in the complex of a propellane derivative with a pair of SnH_3_F molecules.^89^

**Figure 4:**
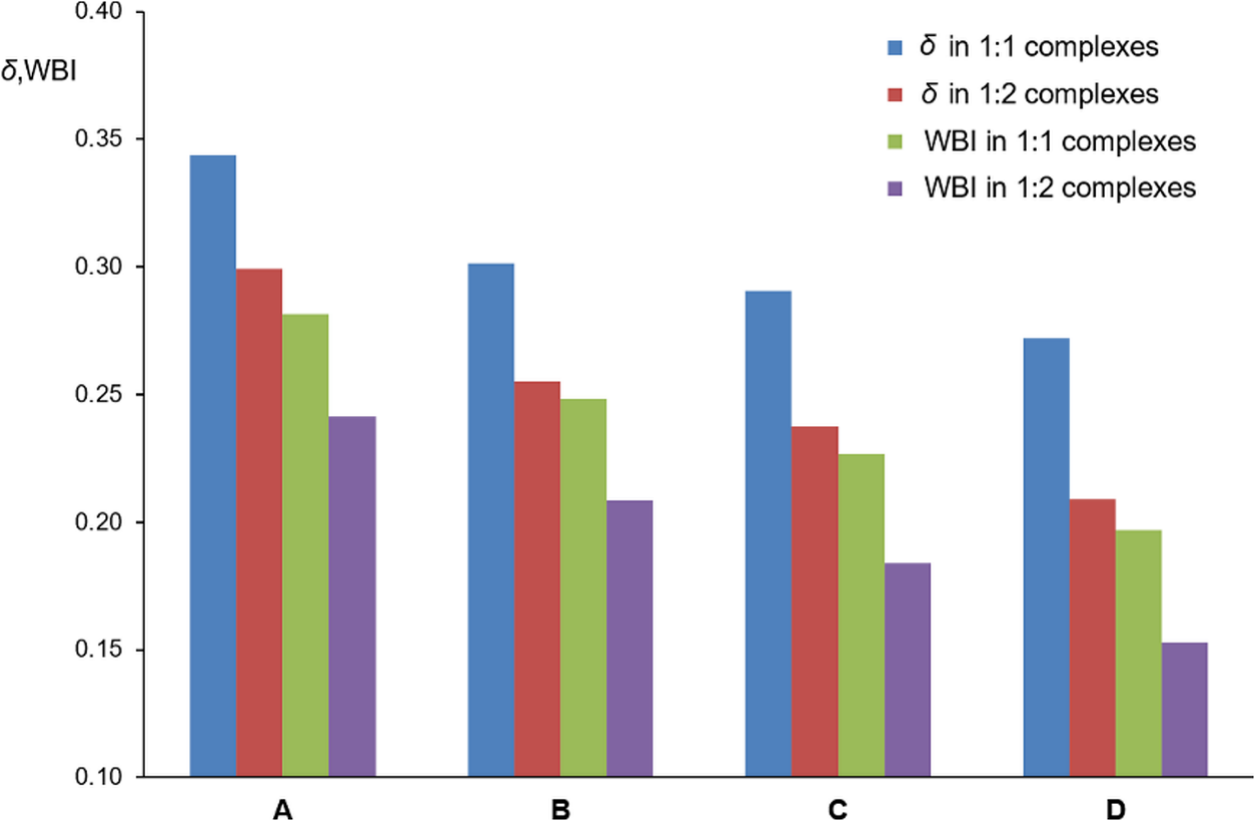
Variations in the delocalization index and the Wiberg bond index between the atoms of N→Sn (δ and WBI, in au) among the 1:1 and 1:2 complexes of **1** and **A**–**D**.

The weakening of the pairwise azine-SnX_4_ interaction has an effect on its fundamental physical nature: the percentage share of LMOEDA dispersion in the pairwise attraction grows while the percentage of the polarization component decreases (Table 5). The percentage of LMOEDA electrostatic energy also tends to decrease upon the formation of all the 1:2 complexes except those coordinating two molecules of **A**. For **1A**_2_–**1D**_2_ the sum of their electrostatic and polarization components counterbalances their destabilizing exchange-repulsion component, but for weaker pairwise azine-SnX_4_ interactions (e.g., **4D**_2_ and **5D**_2_, Table S7) the addition of their dispersion component is needed to obtain the negative sign of the corresponding *ε*_int_(azine,SnX_4_) energies.

**Table 5:** LMOEDA Components (in kJ mol^–1^) of Each Pairwise Interaction between the Molecular Fragments of Azine and SnX_4_ in the 1:1 and 1:2 Complexes of **1** and **A**–**D** ^a^

complex	ε_elst_ (%ε_elst_)	ε_pol_ (%ε_pol_)	ε_disp_ (%ε_disp_)	ε_exch‑rep_
**1A**	–238.2 (51.0)	–181.3 (38.8)	–47.8 (10.2)	295.8
**1A**_2_	–219.0 (51.3)	–161.2 (37.8)	–46.5 (10.9)	268.5
**1B**	–194.6 (48.3)	–154.8 (38.4)	–53.7 (13.3)	290.3
**1B**_2_	–172.0 (48.2)	–132.0 (37.1)	–52.0 (14.7)	255.6
**1C**	–179.1 (47.4)	–142.8 (37.8)	–56.0 (14.8)	284.5
**1C**_2_	–151.7 (47.3)	–115.4 (36.0)	–53.6 (16.7)	241.9
**1D**	–157.8 (46.3)	–124.1 (36.4)	–59.2 (17.4)	271.0
**1D**_2_	–125.2 (45.9)	–92.7 (33.9)	–55.2 (20.2)	217.0

a The percentage share of each attractive component with respect to the pairwise attraction is given in parentheses.

Another manifestation of cooperative effects in the studied inverse coordination complexes is the magnitude and nature of the three-body nonadditive contribution to *E*_int_. As it was mentioned before, this contribution is destabilizing, and therefore, the interaction between the three molecular fragments is anticooperative.^90^ This is in line with the negative cooperativity toward two pairwise azine-SnX_4_ interactions diminishing one another. Although the ε_int_(azine,SnX_4_,SnX_4_^′^) contribution is usually small, it is interesting to examine its fundamental physical nature. To that end, ε_int_(azine,SnX_4_,SnX_4_^′^) was subject to the LMOEDA partitioning and the calculated components are listed in Table 6 for the complexes with the diazine centers (results for the remaining complexes are given in Table S15). It is evident from this table that the three-body contribution is mainly determined by the polarization energy (ε_pol_) while the dispersion (ε_disp_) and exchange-repulsion (ε_exch‑rep_) components are often fairly close to zero, and thus roughly additive. The electrostatic component is not listed because it is strictly pairwise additive by definition.^63^ The destabilizing ε_pol_ component can be crudely rationalized by considering the polarization of one molecular fragment by another fragment as an unfavorable effect on the interaction of the first fragment with the third molecular fragment. The leading role of polarization energy in the three-body nonadditive contribution to the total interaction was previously reported for both model and biologically relevant ternary systems bound by noncovalent interactions such as hydrogen and halogen bonds.^91,92^

**Table 6:** LMOEDA Components (in kJ mol^–1^) of the Three-Body Contribution to *E*_int_ for the Complexes of Two SnX_4_ Molecules Coordinated to Pyrimidine or Pyrazine

complex	ε_pol_	ε_disp_	ε_exch‑rep_
**1A**_2_	26.0	2.5	–2.1
**1B**_2_	18.1	1.6	–2.4
**1C**_2_	15.8	1.1	–3.1
**1D**_2_	10.4	1.1	–4.3
**2A**_2_	22.0	1.2	–1.3
**2B**_2_	15.5	0.3	–0.8
**2C**_2_	12.4	0.0	–0.5
**2D**_2_	8.7	–0.2	–0.3

To have a full understanding of the negative cooperativity in the studied inverse coordination complexes, it is necessary to establish its origin. The interactions between the Lewis basic sites on the azine center and the Lewis acidic sites of 2SnX_4_ lead to a specific distribution of the electron charge among the molecular fragments in the 1:2 complexes. The azine center loses part of its electron charge while the fragments of **A**–**D** acquire an ancillary charge. The QTAIM and NPA analyses of the electron charge distributed within the complexes reveal that the azine center of the 1:2 complexes carries a smaller amount of the electron charge than the corresponding azine fragment of the 1:1 complexes (Figure 5, Table S14). In other words, two SnX_4_ fragments compete for the electron charge of the azine center in the 1:2 complexes. Consequently, the N-donor sites of the azine center in these complexes are characterized by less negative atomic charges (Figure 5). Since the N→Sn bonds of the studied complexes were characterized as largely ionic, the less negative atomic charge on the N-donors results in a weaker electrostatic interaction and lower capacity for possible polarization and charge transfer. Such effects were indeed reported earlier in this section when the LMOEDA components of individual pairwise azine-SnX_4_ interactions in the 1:2 complexes were compared with the LMOEDA results calculated for the interaction between the molecular fragments of the corresponding 1:1 complexes (Table 5).

**Figure 5:**
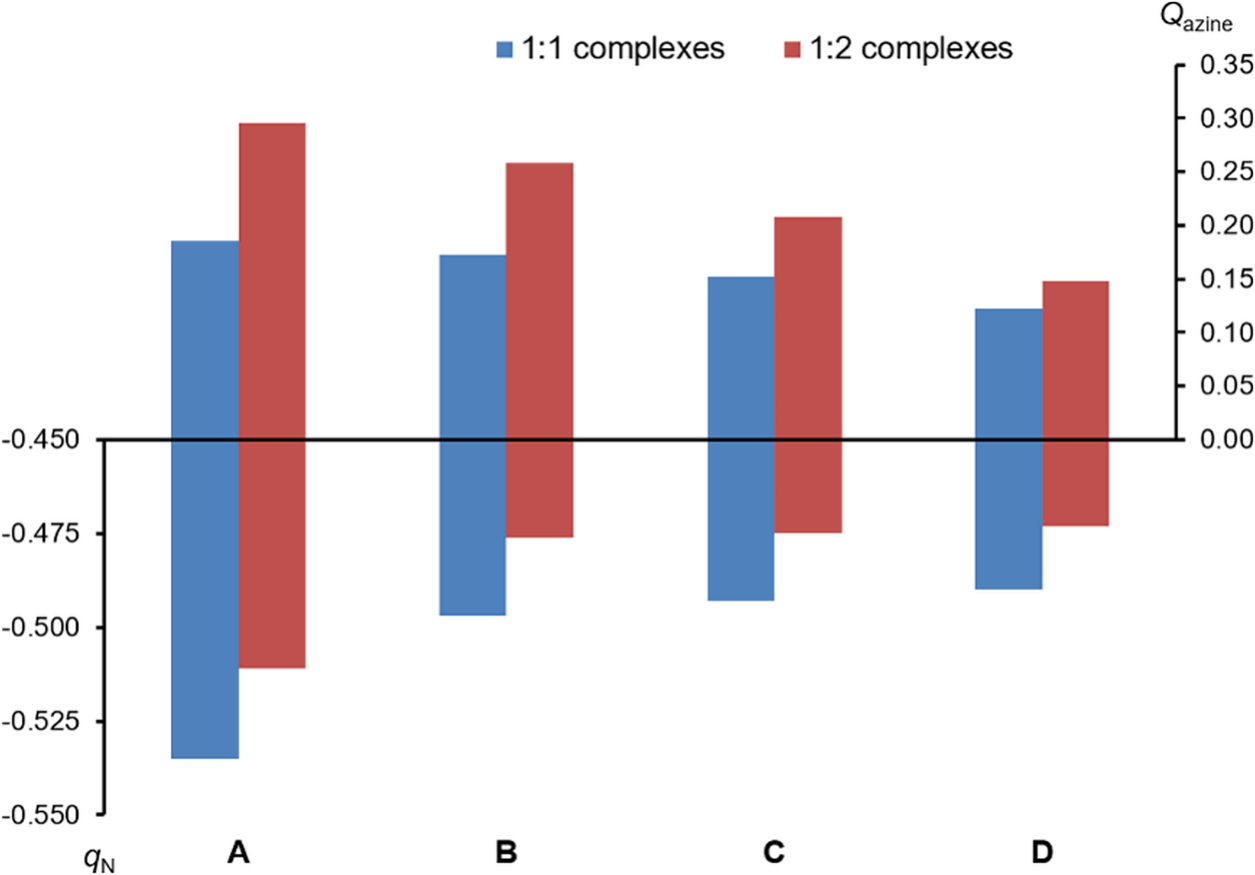
Variations in the NPA electron charge acquired by the azine center (*Q*_azine_, in au) and its N-donor site involved in N→Sn (*q*_N_, in au) among the 1:1 and 1:2 complexes of **1** and **A**–**D**.

## Conclusions

This work has presented the first computational quantum chemical investigation of cooperative effects between two N→Sn coordinate bonds in a series of model inverse coordination complexes inspired by the CSD search for a rare structural motif featuring a single aromatic azine center with two coordinated Sn­(IV) acceptors. The structure and stability of the resulting 1:2 complexes demonstrate a characteristic dependence on the kind of halogen terminal ligands in SnX_4_, as well as on the number and positioning of N atoms in the ring of azine centers. Such factors act on N→Sn bonds in essentially the same way for both conventional and inverse coordination complexes, and they can be explained in terms of Lewis acidity and basicity of Sn-acceptor and N-donor sites. The introduction of another SnX_4_ molecule into the 1:1 complex results in an elongation of N→Sn bonds and a decrease in their strength, as indicated by the weakened pairwise interactions between the azine fragment and each coordinated SnX_4_ fragment in the 1:2 complexes. The formation of two N→Sn bonds with a single inverse coordination center also leads to a certain change in the nature of these bonds: they became less covalent in character and individual pairwise azine-SnX_4_ interactions show a higher percentage share of dispersive attraction. The coordination of the second SnX_4_ molecule reduces the amount of the electron charge localized on the azine center, its N-donors in particular, which weakens their interactions with the SnX_4_ fragments. Furthermore, the many-body analysis of the total interaction energy in the 1:2 complexes reveals the destabilizing three-body contribution yet its magnitude is one or even two orders smaller than the total interaction energy. Thus, negative cooperativity occurs for the strength of N→Sn bonds in the studied inverse coordination complexes.

Even though the aforementioned conclusions have been derived from computations for the inverse coordination complexes in their gas-phase structures, which do not reflect certain effects occurring in the crystalline phase, the presence of the second SnX_4_ molecule is very likely to affect the coordinate N→Sn bonds to a greater extent than crystal packing,^93^ and therefore, we believe these conclusions expand our understanding of how two N→Sn bonds influence one another in the crystal structures of azine-centered ditin­(IV) inverse coordination complexes. Moreover, in view of the significance of aromatic azines in coordination chemistry and their novel role as inverse coordination centers in metal complexes, these conclusions may contribute to developing strategies for the structural design of azine centers to fine-tune the strength of their coordinate bonds with metallic acceptors.

## Supplementary Material



## Notes

Further computational details; validation of computational methodology; Lewis properties of tin­(IV) halides and azines; 1:1 conventional coordination complexes; nature of N→Sn bonds; and additional tables and figures (PDF)
