# Supplementary material for: Cooperative Effects in the Inverse Coordination Complexes of Aromatic Azines and Tin(IV) Halides
Source: J Phys Chem A. 2026 Jul 5;130(28):5544–54. doi: 10.1021/acs.jpca.6c03412 (PMC13383829; doi:10.1021/acs.jpca.6c03412)
Supplement: Supplementary file 1 [file jp6c03412_si_001.pdf]

Supporting Information

for

Cooperative Effects in the Inverse Coordination Complexes of Aromatic Azines and Tin(IV) Halides

by

Piotr Matczak*

Department of Physical Chemistry, Faculty of Chemistry, University of Lodz,

Pomorska 163/165, 90236 Lodz, Poland

*Corresponding author: piotr.matczak@chemia.uni.lodz.pl

This document contains the following supplementary sections:

S1. Further computational details

S2. Validation of the computational methodology used in this work

S3. Lewis acidity of tin(IV) halides and Lewis basicity of azines

S4. 1:1 Conventional coordination complexes of azines and tin(IV) halides

S5. Nature of N→Sn bonds

S6. Additional tables and figures

References

Section S1. Further computational details

Geometry optimizations and subsequent harmonic vibrational frequency calculations for the isolated molecules of **1**–**5**, **A**–**D**, and their complexes in closed-shell singlet ground states were carried out at the BP-D/aug-cc-pVTZ(-PP) level of theory.[1-6] These calculations took advantage of the resolution-of-the-identity approach for computing the electronic Coulomb interaction (RI-*J*),[7,8] hence the corresponding auxiliary basis set was used.[9] The BP-D method was composed of the Becke 88 exchange functional,[1] the Perdew 86 correlation functional[2] and the corresponding Grimme's DFT-D dispersion correction.[3] The BP-D density functional was combined with the aug-cc-pVTZ basis set for the atoms of H, C, N, F, and Cl.[4] For the atoms of Sn, Br and, I, their core electrons were replaced by the corresponding energy-consistent Stuttgart/Cologne MDF pseudopotential (PP),[5] and the resulting aug-cc-pVTZ-PP basis set[6] was assigned to these atoms. The DFT calculations were performed with either the 'm4' multiple grid or a standard dense grid for numerical integration. For each studied complex, the procedure of its geometry optimization sought a local energy minimum until tight convergence criteria (that is, changes in energy < 10^{-6}, RMS gradient < 10^{-5} and RMS geometrical displacement < $4\cdot10^{-5}$ a.u.) were fulfilled. The optimized geometrical structures of all studied complexes are available free of charge via the online repository Zenodo. The XYZ files containing these geometries and the corresponding BP-D/aug-cc-pVTZ(-PP) total electronic energies and zero-point vibrational energies can be downloaded from https://doi.org/10.5281/zenodo.15879812.

The BP-D/aug-cc-pVTZ(-PP) level of theory was used to characterize the formation of each complex by means of its complexation energy (E_{complex}) that was defined as

$$E_{\mathrm{complex}} = E_{\mathrm{int}} + E_{\mathrm{def}} + \Delta \mathrm{ZPVE} \quad \text{(S1)}$$

where E_{int} is the total interaction energy between all molecular fragments in the complex, E_{def} is the so-called deformation energy needed to reorganize the isolated molecules to their geometries observed in the complex, and ΔZPVE denotes the difference in the unscaled zero-point vibrational energies of the complex and the isolated molecules.

Spin-orbit relativistic effects on the total interaction energy in the complexes optimized at the BP-D/aug-cc-pVTZ(-PP) level were estimated using the BP-D density functional with its two-component orbitals treated within the exact two-component (X2C) method.[10,11] The two-component form of orbitals allows for handling spin-orbit coupling (SOC). In general, relativistic X2C calculations require an all-electron orbital basis set. In this work, the all-electron aug-cc-pVTZ-DK basis set[12,13] was used in two-component X2C-BP-D single-point energy calculations. The Kramers invariant density functional formalism and the direct inversion in the iterative subspace (DIIS) scheme for complex Fock operators were activated for these calculations.

The coupled-cluster method incorporating single, double and perturbative triple excitations (CCSD(T))[14] was combined with an extrapolation to the complete basis set (CBS) limit in order to provide an accurate estimation of interaction energies in the studied complexes. However, canonical CCSD(T)/CBS calculations are generally very demanding on computational resources, and in our case, performing such calculations was infeasible for part of the complexes. Because our computational protocol assumed a uniform computational method for all complexes, a less computationally expensive approach to CCSD(T)/CBS energies was required. In consequence, the pair natural orbital (PNO) formalism[15,16] was adopted to reduce the computational cost of CCSD(T) calculations. Additionally, the resolution-of-the-identity (RI) approximation, together with the needed auxiliary basis set,[17,18] and the frozen core (FC) approximation in the treatment of electron correlation were employed to speed up these calculations. The CBS limit of the total electronic energies for the resulting CCSD(T) approach ($E_{tot}^{CCSD(T)/CBS}$) was defined as a sum of the extrapolated Hartree-Fock (HF) energy ($E_{tot}^{HF/CBS}$)

and the extrapolated CCSD(T) correlation energy ($E_{\text{corr}}^{\text{CCSD(T)/CBS}}$). The $E_{\text{tot}}^{\text{HF/CBS}}$ energy was estimated using the HF energies obtained from the HF/aug-cc-pVTZ(-PP) and HF/aug-cc-pVQZ(-PP) single-point energy calculations and then inserted into the following extrapolation formula:[19]

$$E_{\text{tot}}^{\text{HF/CBS}} = \frac{E_{\text{tot}}^{\text{HF/aug-cc-pVTZ(-PP)}} e^{\beta x_3} - E_{\text{tot}}^{\text{HF/aug-cc-pVQZ(-PP)}} e^{\beta x_4}}{e^{\beta x_3} - e^{\beta x_4}} \quad \text{(S2)}$$

where $\beta = 1.62$, $x_3 = 2.96$ and $x_4 = 3.87$.[20] The extrapolation of the CCSD(T) correlation energy to the CBS limit was achieved by using the correlation energies from CCSD(T)/aug-cc-pVTZ(-PP) and CCSD(T)/aug-cc-pVQZ(-PP) single-point energy calculations:[21]

$$E_{\text{corr}}^{\text{CCSD(T)/CBS}} = \frac{x_4^3}{x_4^3 - x_3^3} E_{\text{corr}}^{\text{CCSD(T)/aug-cc-pVQZ(-PP)}} - \frac{x_3^3}{x_4^3 - x_3^3} E_{\text{corr}}^{\text{CCSD(T)/aug-cc-pVTZ(-PP)}} \quad \text{(S3)}$$

where x_3 and x_4 were equal to 2.71 and 3.68, respectively.[22]

The many-body analysis was performed for the total interaction energies calculated at the aforementioned CCSD(T)/CBS level in order to estimate the strength of individual interactions between the molecular fragments in the studied complexes, that is, between the azine and SnX_4 fragments. According to this analysis, the total interaction energy (E_{int}) of complexes possessing three molecular fragments can be partitioned into two- and three-body contributions (ε_{int}). For our complexes of azines and $2SnX_4$, their E_{int} was partitioned in the following contributions:

$$E_{\text{int}} = \varepsilon_{\text{int}}(\text{azine,SnX}_4) + \varepsilon_{\text{int}}(\text{azine,SnX}_4') + \varepsilon_{\text{int}}(\text{SnX}_4\text{,SnX}_4') + \varepsilon_{\text{int}}(\text{azine,SnX}_4\text{, SnX}_4') \quad \text{(S4)}$$

where the first three terms on the right-hand side were two-body (or pairwise) contributions and the fourth was the three-body contribution. Each two-body contribution described the interaction in the pair of molecular fragments in a complex. In Eq. S4 the two SnX_4 fragments were distinguished one from another (that is, SnX_4

and SnX_4'). However, these fragments in part of the studied complexes were arranged symmetrically with respect to the azine fragment, and therefore, the ε_{int} contributions between azine and each SnX_4 fragment were identical. As a result, one of the first two terms on the right-hand side in Eq. S4 could be just doubled.

$$E_{int} = 2\varepsilon_{int}(\text{azine,SnX}_4) + \varepsilon_{int}(\text{SnX}_4,\text{SnX}_4') + \varepsilon_{int}(\text{azine, SnX}_4,\text{SnX}_4') \qquad \text{(S5)}$$

The two-body contributions were obtained by subtracting the total energies of two molecular fragments (E_{tot}) from the total energy of the fragment containing these two molecules.

$$\varepsilon_{int}(\text{azine,SnX}_4) = E_{tot}(\text{azine,SnX}_4) - E_{tot}(\text{azine}) - E_{tot}(\text{SnX}_4) \qquad \text{(S6)}$$

$$\varepsilon_{int}(\text{SnX}_4,\text{SnX}_4') = E_{tot}(\text{SnX}_4,\text{SnX}_4') - E_{tot}(\text{SnX}_4) - E_{tot}(\text{SnX}_4') \qquad \text{(S7)}$$

All the fragments adopted their geometries observed in the optimized complexes of azines and $2SnX_4$. The three-body contribution was calculated using the total energy of the whole complex and the total energies of the constituent fragments containing one and two molecules.

$$\varepsilon_{int}(\text{azine,SnX}_4,\text{SnX}_4') = E_{tot}(\text{azine,SnX}_4,\text{SnX}_4') - E_{tot}(\text{azine,SnX}_4)$$

$$- E_{tot}(\text{azine,SnX}_4') - E_{tot}(\text{SnX}_4,\text{SnX}_4') + E_{tot}(\text{azine}) + E_{tot}(\text{SnX}_4) + E_{tot}(\text{SnX}_4') \qquad \text{(S8)}$$

The total energies in Eqs. S6–S8 were calculated at the CCSD(T)/CBS level defined by Eqs. S2 and S3. In Eqs. S6–S8 the basis set describing the whole complex (the so-called supermolecular or trimer-center basis set scheme) was used in order to remove the basis-set superposition error from ε_{int} values. In other words, the counterpoise method was used to eliminate the basis set superposition error in E_{int}.[23]

The interaction energies between the molecular fragments of the studied complexes were also examined using the localized molecular orbital energy decomposition analysis (LMOEDA)[24] to reveal their fundamental physical nature. According to the LMOEDA scheme, these energies were partitioned into four major components having a clear physical interpretation. Electrostatic interactions such as nuclear-nuclear, 1-electron

and 2-electrons electrostatic interactions, were grouped into the electrostatic component ε_{elst}. Exchange and repulsion energies were joined together into the exchange-repulsion component $\varepsilon_{exch\text{-}rep}$. The polarization component ε_{pol} included orbital relaxation effects. Finally, the dispersion component ε_{disp} was composed of the Kohn–Sham correlation energy and the dispersion energy calculated using Grimme's DFT-D empirical dispersion correction combined with the density functional applied. The LMOEDA scheme was applied to the interaction energies calculated using the r^2SCAN-D density functional[25-27] and the aug-cc-pVTZ(-PP) basis set within the supermolecular scheme.

The electron density (ρ) generated from the wave function of each studied complex in its geometry optimized at the BP-D/aug-cc-pVTZ(-PP) level of theory was analyzed using the quantum theory of atoms in molecules (QTAIM).[28] This molecular wave function was calculated at the BP-D/aug-cc-pVTZ(-PP) level. The QTAIM analysis yielded a set of the critical points of ρ (that is, the spatial points where the first derivative of ρ vanishes) for each complex. The number and type of these critical points always fulfilled the Poincaré-Hopf relationship. Automatic QTAIM basin integration methods (Proaim or 1st order Promega) with a very fine spacing between interatomic surface paths were employed in the QTAIM analysis carried out with the AIMAll 19.10.12 program.[29] The numerical accuracy of integration processes in the QTAIM calculations was monitored by (*i*) keeping the atomic integral of the Laplacian of ρ below $5\cdot10^{-3}$ a.u. in each atomic basin and (*ii*) ensuring that the absolute error in the summation of electron populations in all atomic basins of a given complex is below $5\cdot10^{-3}$ a.u. relative to the number of electrons in this complex.

The natural bond orbital (NBO) analysis[30] of the studied complexes operated on their BP-D/aug-cc-pVTZ(-PP) wave functions.

The electron charge acquired by the azine fragment in the studied complexes was calculated as the sum of partial charges on the atoms constituting the fragment. Both QTAIM and natural population analysis (NPA)[31] partial atomic charges were used.

In addition to the 1:2 inverse coordination complexes, a series of 1:1 conventional coordination complexes containing one azine molecule **1**–**5** coordinated to the SnX_4 molecule **A**–**D** via a N→Sn bond was also considered in this work. Both 1:2 and 1:1 complexes were studied using the same, described above, computational methodology.

Geometry optimizations and the subsequent calculations of harmonic vibrational frequencies, complexation and interaction energies, and LMOEDA components were carried out using the TURBOMOLE 7.7 program.[32] Extended wavefunction files (.wfx files) were provided by the Gaussian 09 D.01 program[33] in a series of single-point energy calculations for complexes in their optimized geometries. The QTAIM implementation available in AIMAll 19.10.12[29] was used. The NBO analysis was performed with the NBO 6.0 program.[34] The analysis of the molecular electrostatic potential was performed with the aid of the Multiwfn 3.8 program.[35]

The computational quantum-chemical part of this work was supplemented by a survey of X-ray diffraction crystal structures retrieved from the Cambridge Structural Database (CSD version 6.0, CSD updates up to April 2025).[36] The ConQuest program[37] was used as a search interface to the CSD. In the CSD searches only crystal structures with no disorder and errors, and R factors of less than 0.1 were taken into account.

Section S2. Validation of the computational methodology used in this work

In this work the geometrical structures of all studied complexes were fully optimized at the BP-D/aug-cc-pVTZ(-PP) level of theory. The choice of the dispersion-corrected BP-D density functional was justified by its good performance in predicting the geometries of tin complexes formed through N→Sn coordination.[38-42] Aside from the use of this functional in previous studies, an extra test of BP-D performance in predicting the geometrical structures of the complexes studied in this work was carried out. Complexes of 1,3,5-triazine (**4**) with two SnX_4 molecules (**A**–**D**) were selected for additional geometry optimizations at the ωB97X/aug-cc-pVTZ(-PP) and MP2/aug-cc-pVTZ(-PP) levels of theory. The ωB97X functional is another DFT method previously used for calculating geometries of tin complexes[38,39,42,43] while the MP2 method was often employed in complexes with tetrel bonds between N and Sn.[44-46] The geometries of complexes $\mathbf{4A_2}$–$\mathbf{4D_2}$ optimized at the two DFT levels were compared with those produced by the much more computationally expensive post-Hartree-Fock method (that is, MP2).

Coordinate N→Sn bond lengths (*d*) calculated for $\mathbf{4A_2}$–$\mathbf{4D_2}$ at the BP-D/aug-cc-pVTZ(-PP), ωB97X/aug-cc-pVTZ(-PP) and MP2/aug-cc-pVTZ(-PP) levels are presented in Table S1. From this table, it is evident that all three methods produce the same increasing trend in *d* values while the X atoms of $2SnX_4$ become heavier and heavier. The two DFT levels tend to overestimate the N→Sn bond lengths, as compared to the *d* values yielded by the MP2 method. The BP-D functional overestimates the N→Sn bond lengths in $\mathbf{4A_2}$–$\mathbf{4D_2}$ by 11.5 pm at most, while the ωB97X functional predicts much longer N→Sn bond lengths (by as much as 77.4 pm). The significantly better performance of BP-D is due to its dispersion correction. The inclusion of this correction turns out to be critical to predicting reliable geometries for complexes featuring $2SnX_4$ weakly-bound with the azine center (in our case, $\mathbf{4C_2}$ and $\mathbf{4D_2}$). To sum up, this test confirmed that the BP-D/aug-cc-pVTZ(-PP) level was adequate to provide the geometrical structures of the studied complexes with reasonable accuracy.

Table S1. Coordinate N→Sn bond lengths (d, in pm) calculated for $\mathbf{4A_2}$–$\mathbf{4D_2}$ at various levels of theory.

Complex	d		
	BP-D/aug-cc-pVTZ(-PP)	ωB97X/aug-cc-pVTZ(-PP)	MP2/aug-cc-pVTZ(-PP)
$\mathbf{4A_2}$	237.4	234.3	230.7
$\mathbf{4B_2}$	255.4	272.9	246.9
$\mathbf{4C_2}$	264.8	311.7	254.8
$\mathbf{4D_2}$	278.2	344.1	266.7

Interaction energies and their many-body analysis for all studied complexes were calculated at the CCSD(T)/CBS level of theory. This level is widely regarded as the contemporary "gold standard" of quantum chemistry for the calculations of interaction energies.[47] It has also been reported that CCSD(T)/CBS is capable of delivering very accurate results for coordination complexes and is often used as a computational reference for benchmarking the performance of density functionals.[48,49] This suggests that the CCSD(T)/CBS calculations of interaction energies between molecular fragments in the complexes of azines and tin(IV) halides are expected to yield highly accurate results. However, the analysis of interaction energies in terms of LMOEDA components was not feasible to be done at the CCSD(T)/CBS level due to the limited implementation of the LMOEDA method in TURBOMOLE. In consequence, the interaction energies obtained at a DFT level had to be adopted in the LMOEDA analysis. Choice of an appropriate DFT level for this analysis based on a comparison of DFT interaction energies with the reference results obtained from CCSD(T)/CBS. In addition to the BP-D functional, two other density functionals, namely M06-2X and r^2SCAN-D, were combined with the aug-cc-pVTZ(-PP) basis set in order to test the performance of DFT in the reproduction of CCSD(T)/CBS interaction energies for the complexes of azines and $2SnX_4$. The M06-2X density functional[50] was selected due to its previous use for calculating interaction energies in tin complexes.[51] The inclusion of r^2SCAN-D[52] was motivated by its nonempirical nature and exceptional performance in predicting the energetics of intermolecular interactions. This functional was proposed quite recently and was combined with the latest generation of semi-classical London dispersion corrections (D4).[27]

For 28 complexes of azines and $2SnX_4$ (**1A**$_2$–**5D**$_2$), their total interaction energies (E_{int}) were calculated at the BP-D/aug-cc-pVTZ(-PP), M06-2X/aug-cc-pVTZ(-PP) and r^{2}SCAN-D/aug-cc-pVTZ(-PP) levels of theory and then compared with the corresponding CCSD(T)/CBS values. This comparison assumed that the complexes always adopted their geometries optimized at the BP-D/aug-cc-pVTZ(-PP) level. The root mean square deviation in the E_{int} values obtained from the DFT methods relative to the reference CCSD(T)/CBS results amounted to 14.3, 6.8 and 6.1 kJ mol^{-1} for BP-D/aug-cc-pVTZ(-PP), M06-2X/aug-cc-pVTZ(-PP) and r^{2}SCAN-D/aug-cc-pVTZ(-PP), respectively. This indicates that r^{2}SCAN-D outperforms the other two functionals in reproducing the CCSD(T)/CBS interaction energies of the complexes of azines with $2SnX_4$. Therefore, the LMOEDA analysis in this work was carried out for the interaction energies obtained from r^{2}SCAN-D/aug-cc-pVTZ(-PP).

Section S3. **Lewis acidity of tin(IV) halides and Lewis basicity of azines**

The Lewis acidity of tin(IV) halides **A**–**D** and the Lewis basicity of azines **1**–**5** were estimated using reactivity indices defined within the framework of conceptual DFT. The Lewis acidity of **A**–**D** was related to the electrophilicity of Sn in SnX_4 whereas the Lewis basicity of **1**–**5** was associated with the nucleophilicity of N in the azines. In addition to the reactivity indices, descriptors based on the electrostatic potential on the molecular surface were also used to estimate the reactivity of **A**–**D** and **1**–**5**.

The electrophilicity of the Sn atom in the isolated molecules of **A**–**D** was expressed in terms of the local electrophilicity index condensed to this atom (ω_{Sn}^{+}).[53] This index was based on the global electrophilicity of **A**–**D** (ω), which was defined by the following formula:

$$\omega = \frac{(\mathrm{IP}+\mathrm{EA})^2}{8(\mathrm{IP}-\mathrm{EA})} \qquad \text{(S9)}$$

where IP and EA are the vertical ionization potential and vertical electron affinity, respectively. The ω_{Sn}^{+} index was formulated as the product of the global electrophilicity and the electrophilic Parr function (P_{Sn}^{+}) on the Sn atom.[54]

$$\omega_{\mathrm{Sn}}^{+} = \omega \cdot P_{\mathrm{Sn}}^{+} \qquad \text{(S10)}$$

The P_{Sn}^{+} function (that is, the Parr function for nucleophilic attacks) was calculated as the atomic spin density at the Sn atom of the radical anion of SnX_4. The atomic spin density at the Sn atom was taken from the results of NPA calculations. Another formulation of the ω_{Sn}^{+} index was also used according to Eq. S11.

$$\omega_{\mathrm{Sn}}^{+} = \omega \cdot f_{\mathrm{Sn}}^{+} \qquad \text{(S11)}$$

In the above equation the quantity f_{Sn}^{+} denotes the electrophilic Fukui function on the Sn atom. The electrophilic Fukui function (that is, the Fukui function for nucleophilic attacks)[55] was calculated using a finite difference approach and the electron populations p_{Sn} on the Sn atom in the neutral SnX_4 molecule and in the SnX_4^{-} anion.

$$f_{\mathrm{Sn}}^{+} = p_{\mathrm{Sn}}(\mathrm{SnX}_4^-) - p_{\mathrm{Sn}}(\mathrm{SnX}_4) \tag{S12}$$

The electron populations p_{Sn} were taken from the NPA analysis. Irrespective of which formulation was used, Eq. S10 or Eq. S11, the more positive value of ω_{Sn}^{+} is, the higher reactivity of the tin atom of SnX_4 towards an electron donor (nucleophilic) reagent, the azine center in our case.

For **1**–**5**, the nucleophilicity of their N atoms was assessed using the local nucleophilicity index condensed to these atoms (ω_{N}^{-}). The ω_{N}^{-} index was formulated as the product of the global electrophilicity and the nucleophilic Parr function (P_{N}^{-}) on a given N atom in the azine ring.[54]

$$\omega_{\mathrm{N}}^{-} = \omega \cdot P_{N}^{-} \tag{S13}$$

The P_{N}^{-} function (that is, the Parr function for electrophilic attacks) was calculated as the atomic spin density at a given N atom of the radical cation of azine. The atomic spin density at the N atom was taken from the NPA analysis. Another formulation of the ω_{N}^{-} index was also used according to Eq. S14.[53]

$$\omega_{\mathrm{N}}^{-} = \omega \cdot f_{N}^{-} \tag{S14}$$

The nucleophilic Fukui function f_{N}^{-} (that is, the Fukui function for electrophilic attacks) was calculated using a finite difference approach and the NPA electron populations p_{N} on a given N atom in the neutral azine molecule and its cationic form.

$$f_{\mathrm{N}}^{-} = p_{\mathrm{N}}(\mathrm{azine}) - p_{\mathrm{N}}(\mathrm{azine}^{+}) \tag{S15}$$

The values of ω, ω_{Sn}^{+} and ω_{N}^{-} were computed for the isolated molecules of **A**–**D** and **1**–**5** optimized at the BP-D/aug-cc-VTZ(-PP) level of theory and subsequently the single-point calculations of neutral, cation and anion molecules were performed using BP-D/aug-cc-VTZ(-PP). The analysis of surface electrostatic potential (V_{S}) was also performed for all the isolated molecules in their optimized geometries. In the analysis of V_{S}, an

isodensity contour of 0.001 a.u. was used to search for the maximal and minimal values of V_s in the regions around the Sn atoms of **A**–**D** and the N atoms of **1**–**5** ($V_{s,max}$ and $V_{s,min}$, respectively).

The ω_{Sn}^{+} and $V_{s,max}$ values calculated for **A**–**D** are presented in Table S2. It is evident that the ω_{Sn}^{+} index adopts systematically decreasing values as the X atom becomes less and less electronegative, that is, while going from F down to I. This trend in ω_{Sn}^{+} signals the expected regular decrease of SnX_4 acidity while going from **A** to **D**. Moreover, the $V_{s,max}$ values of SnX_4 continuously decrease with the growing size of halogen atom. Thus, the trends in both ω_{Sn}^{+} and $V_{s,max}$ are in agreement with the intuitive expectations that the Lewis acidity of SnX_4 (or their electron-acceptor strength) diminishes with the decreasing electronegativity of X, that is, in the sequence **A** > **B** > **C** > **D**.

Table S2. Electrophilicity index condensed to the Sn atom (ω_{Sn}^{+}, in kJ mol^{-1}) and the maximal value of the surface electrostatic potential in the region around the Sn atom ($V_{s,max}$, in kJ mol^{-1}) for tin(IV) halides **A**–**D**.

SnX_4	ω_{Sn}^{+} [a]	ω_{Sn}^{+} [b]	$V_{s,max}$
**A**	143.2	155.1	257.0
**B**	112.6	66.6	137.3
**C**	109.1	48.3	102.7
**D**	108.8	27.8	76.3

[a] According to Eq. S10. [b] According to Eq. S11.

The ω_{N}^{-} and $V_{s,min}$ values of **1**–**5** are listed in Table S3. In general, both ω_{N}^{-} and $V_{s,min}$ show a decrease in their absolute values as the number of N atoms in the azine ring grows. Thus, the trends in the values of both ω_{N}^{-} and $V_{s,min}$ support the diminishing Lewis basicity (or the reduced electron-donor strength) in the sequence diazine > triazine > tetrazine. The greater the N atoms of azines display their electron-donor strength, the more strongly they interact with the electrophilic center of SnX_4. In the case of **1** and **2**, the former exhibits somewhat greater absolute values of ω_{N}^{-} and $V_{s,min}$, which indicates an increased donor ability of the N atoms in **1**. It also

justifies the preference of 1,3-coordination over 1,4-coordination for the diazines. Triazine **3** is non-symmetrical, and therefore, its three N atoms differ in their donor strength. This is reflected in the values of ω_N^- and $V_{s,min}$ that vary among the N atoms at positions 1, 2 and 4 in the ring. The ω_N^- values calculated using Eq. S13 indicate that the N atom at position 2 shows higher ability to donate electrons than the N atoms at positions 1 and 4. This is in line with the most negative $V_{s,min}$ value at position 2. It means that the electrophilic center of SnX_4 interacts favorably with the region of the most negative $V_{s,min}$ value, that is, the lone pair of N atom at position 2. These results would also point to the preference of 1,3-coordination over 1,4-coordination. The formulation of ω_N^- in terms of Eq. S14 seems to perform worse than that using Eq. S13. Part of the ω_N^- values derived from Eq. S14 (for **3** and **4**) diverge from the sequence predicted by $V_{s,min}$ and the ω_N^- index formulated by Eq. S13.

Table S3. Nucleophilicity index condensed to the N atom (ω_N^-, in kJ mol^{-1}) and the minimal value of the surface electrostatic potential in the region of the N-atom lone pair ($V_{s,min}$, in kJ mol^{-1}) for azines **1**–**5**.

Azine	ω_N^- [a]	ω_N^- [b]	$V_{s,min}$
**1**	50.6	32.8	-125.8
**2**	42.1	30.7	-119.4
**3**	40.6; 43.1; 33.3 [c]	28.4; 27.6; 28.0 [c]	-132.5; -136.9; -92.2 [c]
**4**	40.0	28.5	-101.3
**5**	39.4	32.8	-98.9

[a] According to Eq. S12. [b] According to Eq. S13. [c] Values for N atoms at positions 1, 2, and 4, respectively.

Section S4. 1:1 Conventional coordination complexes of azines and tin(IV) halides

In the main document of this study, the N→Sn bonds of the 1:2 inverse coordination complexes were compared with the N→Sn bonds of the corresponding 1:1 conventional coordination complexes. These 1:1 complexes were composed of a single azine molecule attached to a single SnX_4 molecule via the N→Sn bond from the N-donor of the azine to the Sn acceptor of tin(IV) halide. In this case the N→Sn bond can be regarded as a typical coordinate bond between the azine ligand and the metal center, and thus, the 1:1 complexes are termed here as conventional coordination complexes. The 1:1 conventional coordination complexes of azines and SnX_4 are denoted as **1A**, **1B** and so on by analogy with the 1:2 inverse coordination complexes.

The complexation of SnX_4 with azines results in the formation of a penta-coordinated Sn(IV) center with the coordination geometry close to a trigonal bipyramid (Figure S1). One of two apical positions in this coordination polyhedron is occupied by the N-donor of azines. The N→Sn bond lengths (*d*) for the 1:1 complexes in their geometries optimized at the BP-D/aug-cc-pVTZ(-PP) level of theory are listed in Table S4. The calculated *d* values are significantly smaller than the sum of N- and Sn-atom van der Waals radii (372 pm) yet larger that the length of a typical Sn–N bond (205 pm). Each series of the 1:1 complexes containing the fixed azine ligand manifests a clear dependence of *d* on the kind of X. The heavier the halogen atom is bonded to Sn, the longer the N→Sn bond is observed. Similarly, the growing number of N atoms in the azine ring results in an elongation of N→Sn bonds.

The negative values of E_{complex} for all 1:1 complexes in Table S4 prove that their formation from the isolated SnX_4 and azine molecules is energetically favorable. The E_{complex} energies are determined mostly by the contribution from E_{int}. The incorporation of spin-orbit relativistic correction into E_{int} weakens the interaction between SnX_4 and the azine ligand to a small degree (by 3.3 kJ mol^{-1} at most). The second most important

contribution to E_{complex} is the E_{def} energy of SnX_4. The E_{def} contribution is destabilizing due to the considerable deformation of SnX_4 geometries from tetrahedral in the isolated molecules to pyramidal in the 1:1 complexes. The magnitudes of both E_{complex} and E_{int} are related to the Lewis acidity of SnX_4. This leads to the halogen dependence assuming the decreasing strength of N→Sn coordination bonds with the growing atomic number of X. Besides, the growing number of N atoms in the azine ring results in a gradual reduction in the stability of the 1:1 complexes and in the strength of the interaction between the SnX_4 and azine fragments of these complexes. The N-donor at position 2 in the ring of 1,2,4-triazine tends to interact with SnX_4 most strongly, while the N-donor at position 4 is characterized by the weakest interaction with SnX_4.

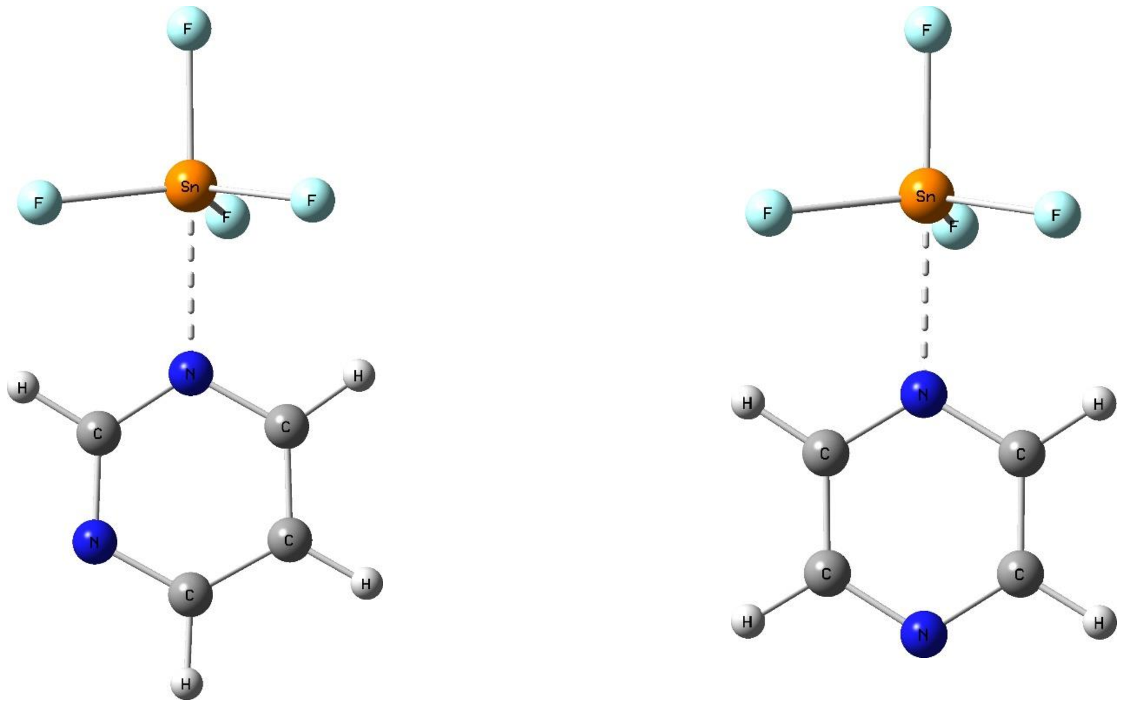

(a) (b)

Figure S1. Optimized lowest-energy geometrical structures of complexes (a) **1A** and (b) **2A**. Hydrogen, carbon, nitrogen, fluorine and tin are colored white, gray, blue, cyan and orange, respectively.

Table S4. Geometrical (in pm) and energetic parameters (in kJ mol^{-1}) for the 1:1 conventional coordination complexes of azines and SnX_4.

Complex	d	$E_{complex}$	E_{int} [a]	E_{def}		ΔZPVE
				azine	SnX_4	
**1A**	230.0	-110.1	-160.1 (-159.0) [-177.5]	3.5	40.6	5.8
**1B**	243.8	-57.0	-107.4 (-106.9) [-118.3]	2.1	43.3	5.1
**1C**	249.3	-47.6	-93.4 (-93.5) [-95.4]	1.7	39.5	4.6
**1D**	257.9	-38.4	-77.7 (-74.4) [-75.4]	1.3	33.9	4.1
**2A**	230.7	-107.3	-155.1 (-154.1) [-172.9]	2.7	39.1	6.0
**2B**	244.6	-55.5	-104.4 (-103.8) [-115.0]	1.6	42.0	5.2
**2C**	250.5	-46.7	-90.5 (-90.6) [-90.5]	1.3	37.8	4.7
**2D**	259.1	-38.0	-75.7 (-72.6) [-71.5]	1.0	32.6	4.1
**3A** [b]	234.2;<br>233.3;<br>233.4	-93.3;<br>-94.9;<br>-92.5	-136.8 (-135.9) [-152.3];<br>-138.9 (-138.0) [-154.1];<br>-133.7 (-132.8) [-145.7]	2.9;<br>3.4;<br>2.7	34.7;<br>34.9;<br>33.0	5.9;<br>5.6;<br>5.4
**3B** [b]	249.0;<br>248.2;<br>249.2	-46.4;<br>-46.4;<br>-44.5	-89.3 (-88.7) [-97.2];<br>-90.1 (-89.6) [-98.8];<br>-84.9 (-84.5) [-92.9]	2.1;<br>2.3;<br>1.5	35.7;<br>36.4;<br>34.5	5.1;<br>5.0;<br>4.5
**3C** [b]	255.4;<br>254.6;<br>256.2	-38.4;<br>-38.0;<br>-37.2	-76.5 (-76.7) [-77.8];<br>-76.9 (-77.0) [-79.1];<br>-72.9 (-73.1) [-71.1]	1.8;<br>2.0;<br>1.3	31.7;<br>32.5;<br>30.4	4.6;<br>4.4;<br>4.0
**3D** [b]	265.5;<br>264.7;<br>269.0	-31.9;<br>-31.0;<br>-30.6	-62.9 (-60.5) [-60.3];<br>-63.0 (-60.6) [-61.1];<br>-58.8 (-56.5) [-60.4]	1.5;<br>1.6;<br>0.8	25.5;<br>26.6;<br>24.0	4.0;<br>3.8;<br>3.4
**4A**	232.6	-96.1	-140.1 (-139.2) [-153.8]	3.7	35.2	5.1
**4B**	247.7	-45.5	-88.6 (-88.2) [-95.6]	2.1	36.5	4.5
**4C**	254.6	-37.5	-75.1 (-75.3) [-74.8]	1.7	32.0	4.0
**4D**	267.4	-30.0	-59.5 (-57.2) [-54.2]	1.1	25.0	3.4
**5A**	237.8	-76.9	-112.9 (-112.2) [-123.6]	2.8	28.0	5.1
**5B**	256.0	-33.6	-67.3 (-66.9) [-72.8]	1.8	27.5	4.4
**5C**	263.2	-27.9	-56.0 (-56.1) [-54.4]	1.6	22.6	3.9
**5D**	277.5	-24.4	-45.0 (-43.5) [-37.1]	1.2	16.2	3.3

[a] Interaction energies including both scalar and spin-orbit relativistic corrections are given in parentheses. Interaction energies obtained from CCSD(T)/CBS are given in square brackets. [b] Results are ordered for N→Sn involving the N atom at position 1, 2 or 4, respectively.

Section S5. Nature of N→Sn bonds

The nature of N→Sn bonds in the studied inverse coordination complexes can be elucidated in terms of several QTAIM parameters based on the electron density (ρ) calculated for the complexes. In general, the QTAIM method predicts that the N and Sn atoms forming each N→Sn bond in the complexes are always linked by a bond path (BP). Figure S2 presents the QTAIM molecular graphs of two exemplary complexes (**1A**$_2$ and **2A**$_2$). As shown in this figure, along each of the BPs, which mark out the lines of concentrated ρ linking pairs of atomic centers, there is a saddle point in ρ, called the (3,-1) critical point or the bond critical point (BCP). From the QTAIM point of view, the presence of a BP and a BCP between two atoms meets the necessary and sufficient condition that these atoms are bonded to one another.[56]

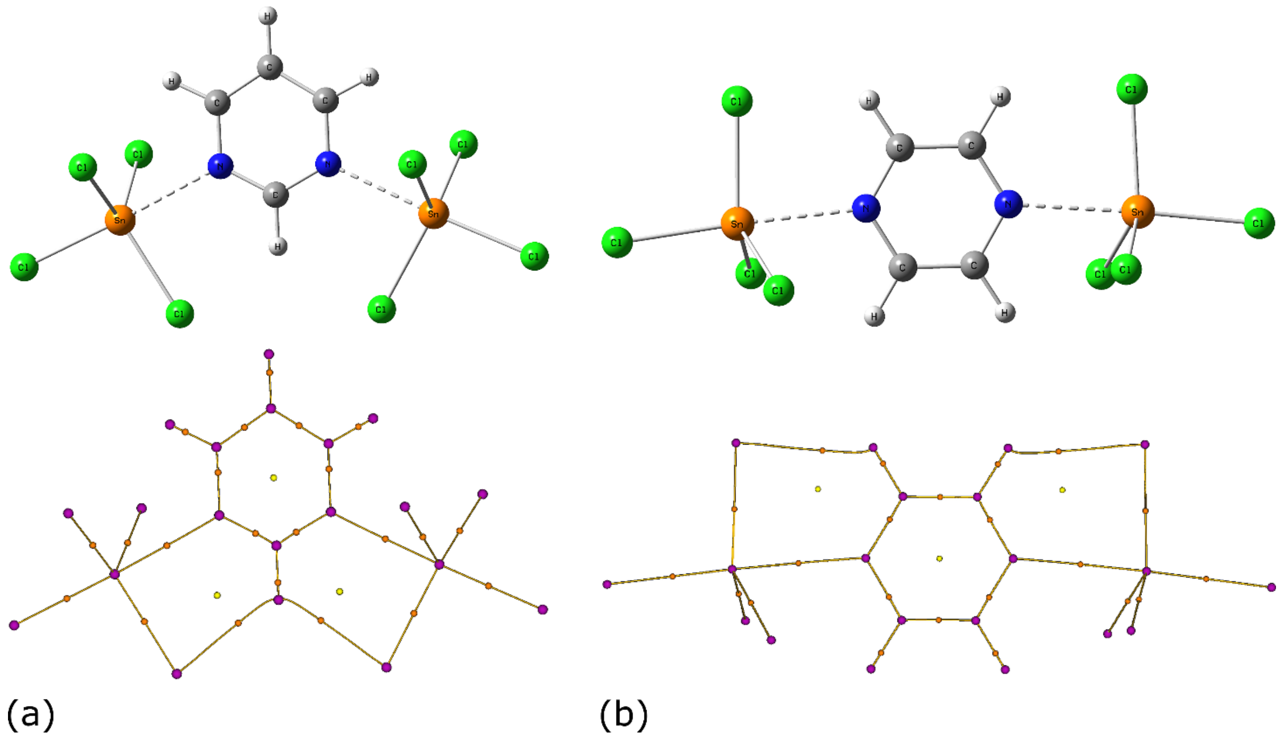

Figure S2. Optimized lowest-energy geometrical structures and QTAIM molecular graphs for complexes (a) **1B**$_2$ and (b) **2B**$_2$. Bond paths are drawn with amber lines. Bond critical points are shown as small orange spheres and ring critical points as small yellow spheres. Nuclear attractor critical points found in the positions of atomic nuclei are colored purple.

Several QTAIM parameters calculated at the BCP of N→Sn are presented in Table S5. The values of ρ at this BCP (ρ_b) are relatively small and these values are greater than 0.05 a.u. only for the complexes containing $2SnF_4$. The Laplacian of ρ at this BCP ($\nabla^2\rho_b$) also adopts relative low and positive values. According to these two parameters, the N→Sn interaction can be categorized as electrostatic (or ionic, or closed shell).[28] On the other hand, the electron potential energy density at the BCP of N→Sn (V_b) predominates over the electron kinetic energy density (G_b), resulting in the negative yet small values of the total electron energy density ($H_b = V_b + G_b$) for all complexes except **3D**$_2$ and **5D**$_2$. The negative sign of H_b indicates the existence of a certain covalent contribution to the N→Sn interaction. Consequently, the ρ_b, $\nabla^2\rho_b$ and H_b parameters of N→Sn determine a mixed character (that is, largely ionic with a covalent component) for the N→Sn interaction in all complexes except **3D**$_2$ and **5D**$_2$. Alternatively, such an interaction is often termed as the intermediate type of interatomic interactions.[57] Furthermore, the mixed character of the N→Sn interaction is also supported by the adimensional ratio of energy densities ($-V_b/G_b$). The values of this ratio fall into the range between 1.0 and 2.0, that is, between typical ionic interactions ($-V_b/G_b < 1.0$) and typical covalent interactions ($-V_b/G_b > 2.0$).[58] In this regard, the QTAIM characteristics of the BCP between N and Sn in these complexes are essentially quite typical of metal-ligand interactions.[59-61] However, QTAIM characteristics underscore an ionic character for the N→Sn bond between the acceptor Sn atom of SnI_4 and the donor N atom at position 4 in the ring of 1,2,4-triazine (**3D**$_2$) and for both N→Sn bonds of **5D**$_2$. The electrostatic character of N→Sn interaction in these two complexes seems to lend support to the alternative elucidation of their formation via the σ-hole interaction resulting in the occurrence of the tetrel bond.[44,62]

Aside from the QTAIM parameters determined at the BCP between N and Sn, the delocalization index (δ) between the interacting N and Sn atoms is another QTAIM parameter providing an insight into the nature of N→Sn interactions. As a general rule, the δ parameter is regarded as a reliable descriptor of covalency of interatomic interactions.[63] The δ values calculated for the studied inverse coordination complexes vary from 0.082 to 0.301 (Table S5). These values are essentially fairly small – smaller than the δ values between

interacting N and Sn atoms in the 1:1 and 1:2 conventional coordination complexes of tin(IV) halides and pyridine, and considerably smaller than the δ value of a typical covalent Sn–N bond (e.g., 0.78 for $Sn(NH_2)_4$).[40] In consequence, the δ values of the studied inverse coordination complexes clearly demonstrate a relatively small covalent component in the N→Sn interaction. The predominant ionic contribution to the N→Sn interaction arises from the oppositely charged atoms forming the N→Sn bond. The QTAIM charges acquired by the N and Sn atoms forming the N→Sn bonds in the studied inverse coordination complexes are listed in Table S6. It is evident from this table that the atomic charges of both atoms involved in the N→Sn interaction are of opposite signs and large in magnitude. This obviously points to the electrostatic interaction as a pivotal driving force for the N→Sn interaction in the studied complexes.

To confirm the conclusions drawn from QTAIM results, an orbital-based perspective on the nature of N→Sn bonds was derived from the NBO method. This method was used to calculate the Wiberg bond index (WBI)[64] and natural population analysis (NPA)[31] charges for the N→Sn bonds of the studied inverse coordination complexes. The WBI values of the N→Sn bonds are appended to Table S5, while the NPA charges on the N and Sn atom forming these bonds are given in Table S6. The WBI values of the studied N→Sn bonds are below 0.25, which is much smaller than the WBI of a typical covalent Sn–N bond (e.g., 0.71 for $Sn(NH_2)_4$). Thus, the WBI values indicates a small share of ordinary covalent interaction in the N→Sn bonds of the studied inverse coordination complexes. This clearly implies the important role of ionic interaction and the occurrence of such a strong electrostatic interaction is indeed manifested in the values of NPA charges for the atoms forming the N→Sn bonds. The N atom is negatively charged, whereas the Sn atom loses a significant amount of its electron charge. The loss of electron charge on Sn is enhanced by the neighboring electronegative terminal halogen ligands. There is a strong correlation between the calculated WBI and δ values of N→Sn for all the inverse coordination complexes – the coefficient of determination (R^2) for the relation between these two quantities amounts to 0.979.

Table S5. QTAIM and NBO parameters (in a.u.) characterizing the N→Sn bonds in the inverse coordination complexes of two SnX_4 molecules attached at various positions to an azine center.

Complex	ρ_b	$\nabla\rho_b$	H_b	$-V_b/G_b$	δ	WBI
1,3-coordination						
**1A**$_2$	0.0610	0.1409	-0.0140	1.28	0.299	0.241
**1B**$_2$	0.0449	0.0933	-0.0072	1.24	0.255	0.208
**1C**$_2$	0.0388	0.0794	-0.0049	1.20	0.238	0.184
**1D**$_2$	0.0310	0.0617	-0.0024	1.13	0.212	0.154
**3A**$_2$ [a]	0.0556; 0.0555	0.1295; 0.1291	-0.0115; -0.0114	1.26; 1.26	0.268; 0.264	0.218; 0.214
**3B**$_2$ [a]	0.0409; 0.0384	0.0868; 0.0819	-0.0057; -0.0047	1.21; 1.19	0.222; 0.218	0.173; 0.186
**3C**$_2$ [a]	0.0371; 0.0316	0.0765; 0.0669	-0.0043; -0.0025	1.18; 1.13	0.218; 0.190	0.174; 0.145
**3D**$_2$ [a]	0.0370; 0.0133	0.0743; 0.0316	-0.0043; 0.0008	1.19; 0.89	0.238; 0.082	0.177; 0.059
**4A**$_2$	0.0568	0.1335	-0.0119	1.26	0.276	0.222
**4B**$_2$	0.0405	0.0865	-0.0054	1.20	0.227	0.185
**4C**$_2$	0.0339	0.0716	-0.0032	1.15	0.206	0.159
**4D**$_2$	0.0267	0.0560	-0.0012	1.08	0.181	0.133
**5A**$_2$	0.0500	0.1174	-0.0089	1.23	0.233	0.191
**5B**$_2$	0.0331	0.0720	-0.0031	1.15	0.176	0.146
**5C**$_2$	0.0297	0.0638	-0.0020	1.11	0.171	0.135
**5D**$_2$	0.0165	0.0375	0.0005	0.94	0.103	0.079
1,4-coordination						
**2A**$_2$	0.0609	0.1386	-0.0141	1.29	0.301	0.242
**2B**$_2$	0.0449	0.0923	-0.0072	1.24	0.256	0.208
**2C**$_2$	0.0396	0.0797	-0.0052	1.21	0.243	0.188
**2D**$_2$	0.0324	0.0646	-0.0028	1.15	0.221	0.160
**3A**$_2$ [b]	0.0562; 0.0562	0.1302; 0.1293	-0.0118; -0.0117	1.27; 1.26	0.274; 0.266	0.220; 0.219
**3B**$_2$ [b]	0.0410; 0.0393	0.0861; 0.0835	-0.0057; -0.0050	1.21; 1.19	0.222; 0.221	0.186; 0.179
**3C**$_2$ [b]	0.0364; 0.0333	0.0752; 0.0698	-0.0041; -0.0030	1.18; 1.15	0.213; 0.202	0.169; 0.155
**3D**$_2$ [b]	0.0332; 0.0148	0.0673; 0.0340	-0.0031; 0.0007	1.15; 0.91	0.214; 0.096	0.160; 0.071
**5A**$_2$	0.0505	0.1182	-0.0091	1.24	0.236	0.194
**5B**$_2$	0.0337	0.0730	-0.0032	1.15	0.180	0.149
**5C**$_2$	0.0297	0.0635	-0.0020	1.11	0.172	0.137
**5D**$_2$	0.0188	0.0417	0.0003	0.97	0.121	0.091

[a] Results are given for N→Sn involving the N atoms at positions 2 and 4, respectively. [b] Results are given for N→Sn involving the N atoms at positions 1 and 4, respectively.

Table S6. QTAIM and NPA charges (in a.u.) of the atoms forming the N→Sn bonds in the inverse coordination complexes of two SnX_4 molecules attached at various positions to an azine center.

Complex	QTAIM		NPA	
	N	Sn	N	Sn
1,3-coordination				
**1A**$_2$	-1.129	2.545	-0.511	2.477
**1B**$_2$	-1.127	1.919	-0.476	1.458
**1C**$_2$	-1.127	1.592	-0.475	1.149
**1D**$_2$	-1.125	1.188	-0.473	0.713
**3A**$_2$ [a]	-0.618; -1.108	2.549; 2.548	-0.289; -0.472	2.482; 2.478
**3B**$_2$ [a]	-0.624; -1.104	1.919; 1.913	-0.269; -0.435	1.458; 1.454
**3C**$_2$ [a]	-0.610; -1.103	1.585; 1.581	-0.265; -0.438	1.141; 1.141
**3D**$_2$ [a]	-0.623; -1.071	1.199; 1.145	-0.277; -0.415	0.719; 0.692
**4A**$_2$	-1.114	2.548	-0.520	2.481
**4B**$_2$	-1.115	1.917	-0.490	1.455
**4C**$_2$	-1.115	1.585	-0.490	1.145
**4D**$_2$	-1.115	1.178	-0.483	0.704
**5A**$_2$	-0.570	2.550	-0.245	2.483
**5B**$_2$	-0.563	1.910	-0.216	1.455
**5C**$_2$	-0.568	1.576	-0.225	1.135
**5D**$_2$	-0.551	1.156	-0.207	0.698
1,4-coordination				
**2A**$_2$	-1.114	2.543	-0.464	2.476
**2B**$_2$	-1.116	1.917	-0.423	1.457
**2C**$_2$	-1.116	1.592	-0.418	1.151
**2D**$_2$	-1.117	1.190	-0.413	0.713
**3A**$_2$ [b]	-0.607; -1.102	2.548; 2.546	-0.239; -0.466	2.481; 2.477
**3B**$_2$ [b]	-0.612; -1.101	1.918; 1.913	-0.218; -0.435	1.459; 1.453
**3C**$_2$ [b]	-0.613; -1.100	1.591; 1.583	-0.216; -0.430	1.154; 1.142
**3D**$_2$ [b]	-0.623; -1.073	1.196; 1.151	-0.223; -0.409	0.725; 0.692
**5A**$_2$	-0.566	2.548	-0.242	2.481
**5B**$_2$	-0.564	1.911	-0.217	1.455
**5C**$_2$	-0.565	1.576	-0.216	1.135
**5D**$_2$	-0.558	1.157	-0.211	0.689

[a] Results are given for N→Sn involving the N atoms at positions 2 and 4, respectively. [b] Results are given for N→Sn involving the N atoms at positions 1 and 4, respectively.

The covalent contribution to the N→Sn interaction decreases as the atomic number of halogen terminal ligands grows in each series of complexes with the azine center fixed. This decrease of covalency is indicated by (*i*) the diminishing values of δ and WBI, (*ii*) the values of $-V_b/G_b$ getting close to 1.0, (*iii*) the negative values of H_b approaching zero as the X terminal ligands proceed down Group 17 from fluorine to iodine.

Having characterized the nature of N→Sn bonds, it is instructive to provide a broader perspective on the overall interaction between the molecular fragments connected via N→Sn in the studied inverse coordination complexes. To that end, the LMOEDA method was used to analyze the pairwise interaction between the azine center and each of the coordinated SnX_4 molecules. More specifically, the two-body interaction energies between the azine fragment and individual SnX_4 fragments in the complexes ($\varepsilon_{\mathrm{int}}(\mathrm{azine}, \mathrm{SnX_4})$ in Eq. S5) were partitioned into four components reflecting the fundamental physical forces acting between these molecular fragments. The LMOEDA components of $\varepsilon_{\mathrm{int}}(\mathrm{azine}, \mathrm{SnX_4})$ calculated using r^2SCAN-D/aug-cc-pVTZ(-PP) are listed in Table S7. As evidenced by $\varepsilon_{\mathrm{elst}}$ in this table, the interaction between the azine and SnX_4 fragments is determined to a great extent by electrostatic forces, providing between 43% and 51% of the pairwise attraction. The importance of electrostatic component in the interaction between two molecular fragments connected by N→Sn is supported by the results of previous computational studies.[40,65] The percentage contribution of $\varepsilon_{\mathrm{elst}}$ in the inverse coordination complexes decreases while X is substituted with heavier and heavier halogens playing the role of terminal ligands. The $\varepsilon_{\mathrm{pol}}$ component originates from the polarization forces acting between the azine center and SnX_4. This component is the second most stabilizing component for all complexes except **3D**$_2$ and **5D**$_2$. The percentage contribution of $\varepsilon_{\mathrm{pol}}$ varies from 25% to 38%. For complexes **3D**$_2$ and **5D**$_2$, their $\varepsilon_{\mathrm{pol}}$ component provides slightly smaller stabilization than their $\varepsilon_{\mathrm{disp}}$ component does. Dispersion forces are responsible for 11% to 33% of the pairwise attraction between the azine and SnX_4 fragments. The $\varepsilon_{\mathrm{disp}}$ component is particularly important for the complexes showing weaker interaction between the azine and SnX_4 fragments. In this case, the exchange-repulsion component $\varepsilon_{\mathrm{exch\text{-}rep}}$ cannot be counterbalanced by the sum of $\varepsilon_{\mathrm{elst}}$ and $\varepsilon_{\mathrm{pol}}$. Thus, the negative value of $\varepsilon_{\mathrm{int}}(\mathrm{azine}, \mathrm{SnX_4})$ occurs only when the $\varepsilon_{\mathrm{disp}}$ component has been included.

Table S7. LMOEDA components (in kJ mol^{-1}) of the pairwise interaction between the molecular fragments of azine and SnX_4 in the inverse coordination complexes of two SnX_4 molecules attached at various positions to the azine center. The percentage of each attractive component with respect to the pairwise attraction is given in parentheses.

Complex	ε_{int} [a]	ε_{elst} (%ε_{elst})	ε_{pol} (%ε_{pol})	ε_{disp} (%ε_{disp})	$\varepsilon_{exch-rep}$
1,3-coordination					
**1A**$_2$	-158.2	-219.0 (51.3)	-161.2 (37.8)	-46.5 (10.9)	268.5
**1B**$_2$	-100.4	-172.0 (48.3)	-132.0 (37.1)	-52.0 (14.6)	255.6
**1C**$_2$	-78.8	-151.7 (47.3)	-115.4 (36.0)	-53.6 (16.7)	241.9
**1D**$_2$	-56.1	-125.2 (45.9)	-92.7 (33.9)	-55.2 (20.2)	217.0
**3A**$_2$ [b]	-140.7; -131.4	-187.8 (51.1); -185.6 (50.4)	-138.6 (37.7); -138.3 (37.6)	-41.3 (11.2); -44.2 (12.0)	227.0; 236.7
**3B**$_2$ [b]	-75.9; -87.6	-138.3 (47.6); -146.0 (48.1)	-103.2 (35.5); -111.6 (36.8)	-49.1 (16.9); -45.8 (15.1)	214.7; 215.8
**3C**$_2$ [b]	-67.5; -56.6	-134.6 (46.9); -117.4 (46.5)	-102.7 (35.8); -84.6 (33.5)	-49.7 (17.3); -50.4 (20.0)	219.6; 195.8
**3D**$_2$ [b]	-56.7; -24.0	-138.6 (46.0); -56.6 (42.8)	-107.3 (35.6); -32.5 (24.6)	-55.2 (18.3); -43.1 (32.6)	244.4; 108.3
**4A**$_2$	-137.4	-196.5 (51.0)	-144.3 (37.5)	-44.6 (11.6)	247.9
**4B**$_2$	-81.0	-149.1 (48.1)	-111.1 (35.9)	-49.5 (16.0)	228.8
**4C**$_2$	-61.4	-128.3 (47.1)	-93.2 (34.2)	-50.9 (18.7)	211.0
**4D**$_2$	-42.6	-106.3 (45.6)	-74.0 (31.8)	-52.5 (22.6)	190.2
**5A**$_2$	-113.8	-155.1 (50.0)	-116.1 (37.4)	-39.2 (12.6)	196.6
**5B**$_2$	-62.8	-109.6 (47.1)	-80.8 (34.7)	-42.2 (18.1)	169.8
**5C**$_2$	-46.3	-101.9 (45.8)	-73.6 (33.1)	-46.8 (21.1)	176.0
**5D**$_2$	-27.4	-59.8 (43.2)	-38.6 (27.8)	-40.2 (29.0)	111.2
1,4-coordination					
**2A**$_2$	-153.2	-211.1 (50.8)	-158.0 (38.0)	-46.5 (11.2)	262.4
**2B**$_2$	-97.1	-165.9 (47.9)	-128.4 (37.1)	-51.9 (15.0)	249.2
**2C**$_2$	-79.2	-149.4 (46.9)	-115.3 (36.2)	-53.6 (16.8)	239.1
**2D**$_2$	-57.7	-127.1 (45.5)	-95.8 (34.3)	-56.2 (20.2)	221.4
**3A**$_2$ [c]	-137.7; -131.4	-183.9 (50.7); -185.5 (50.3)	-137.4 (37.9); -139.3 (37.7)	-41.7 (11.5); -44.3 (12.0)	225.3; 237.8
**3B**$_2$ [c]	-86.2; -76.8	-142.1 (47.7); -139.9 (47.5)	-110.0 (36.9); -105.3 (35.8)	-46.0 (15.4); -49.2 (16.7)	212.0; 217.6
**3C**$_2$ [c]	-70.5; -59.6	-128.5 (46.6); -121.9 (46.5)	-99.5 (36.1); -89.6 (34.2)	-47.6 (17.3); -50.5 (19.3)	205.1; 202.4
**3D**$_2$ [c]	-56.6; -27.5	-121.2 (45.4); -60.3 (43.2)	-94.2 (35.3); -36.1 (25.8)	-51.4 (19.3); -43.2 (30.9)	210.2; 112.1
**5A**$_2$	-113.6	-155.1 (49.8)	-116.8 (37.5)	-39.3 (12.6)	197.7
**5B**$_2$	-63.0	-110.6 (47.1)	-81.8 (34.9)	-42.4 (18.1)	171.9
**5C**$_2$	-47.5	-100.5 (45.8)	-73.0 (33.3)	-45.8 (20.9)	171.8
**5D**$_2$	-28.8	-68.3 (43.4)	-44.9 (28.5)	-44.1 (28.1)	128.5

[a] Interaction energy ε_{int}(azine,SnX_4) defined by Eq. S6. [b] Results are given for N→Sn involving the N atoms at positions 2 and 4, respectively. [c] Results are given for N→Sn involving the N atoms at positions 1 and 4, respectively.

The overall interaction between the azine fragment and the coordinated SnX_4 fragment is obviously in a strong relationship with the strength of the N→Sn bond connecting these fragments. This relationship can be illustrated by comparing $\varepsilon_{int}(\text{azine},\text{SnX}_4)$ with the QTAIM parameters describing the N→Sn bond. For example, a relationship between $\varepsilon_{int}(\text{azine},\text{SnX}_4)$ and $\nabla^2\rho_b$ is shown in Figure S3. From this figure it can be deduced that the increasing strength of the interaction between the azine fragment and individual SnX_4 fragments is associated with the growing values of $\nabla^2\rho$ at the BCP of the N→Sn bonds connecting these fragments.

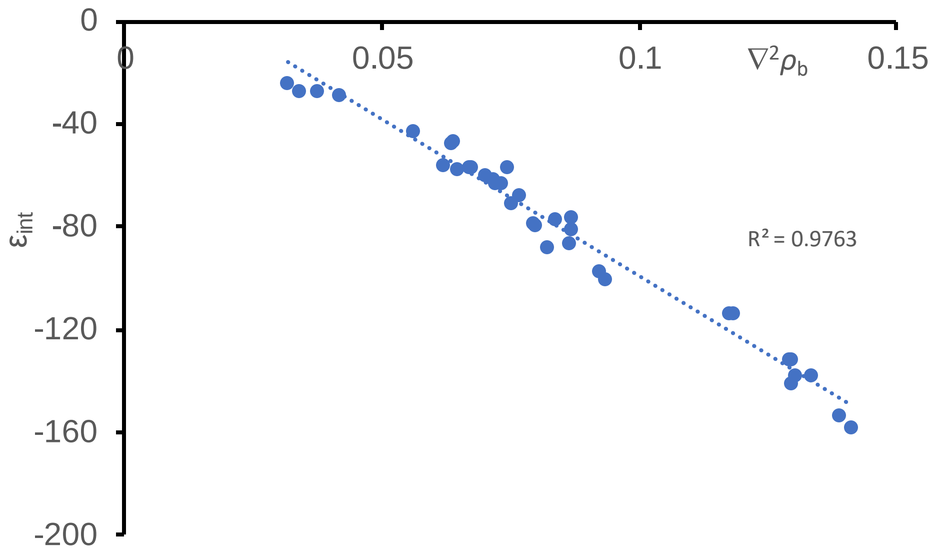

Figure S3. Plot of the pairwise interaction energy between the azine center and the coordinated SnX_4 molecular fragment (ε_{int}(azine,SnX_4), in kJ mol^{-1}) against the Laplacian of the electron density at the bond critical point between N and Sn ($\nabla^2\rho_b$, in a.u.) for the studied inverse coordination complexes. The coefficient of determination (R^2) is also shown for this relation.

Changes in the N→Sn bonds of the inverse coordination complexes upon attaching the second SnX_4 molecule were established relative to the N→Sn bonds of 1:1 conventional coordination complexes of azines and SnX_4. The nature of N→Sn bonds in the 1:1 complexes was characterized using the same methods as those applied to the 1:2 inverse coordination complexes. The QTAIM, NBO and LMOEDA results obtained for the 1:1 complexes are summarized in Tables S8–S10. Roughly speaking, the nature of N→Sn bonds in the 1:1 and 1:2 complexes is similar, that is, of a mixed type between typical ionic and typical covalent interactions. A detailed discussion of differences in the N→Sn bonds of the 1:1 and 1:2 complexes is presented in the main document.

Table S8. QTAIM and NBO parameters (in a.u.) characterizing the N→Sn bond in the 1:1 conventional coordination complexes of azines and SnX_4.

Complex	ρ_b	$\nabla\rho_b$	H_b	$-V_b/G_b$	δ	WBI
**1A**	0.0679	0.1536	-0.0176	1.31	0.344	0.281
**1B**	0.0524	0.1069	-0.0104	1.28	0.301	0.248
**1C**	0.0472	0.0942	-0.0081	1.26	0.291	0.227
**1D**	0.0402	0.0789	-0.0053	1.21	0.272	0.197
**2A**	0.0672	0.1508	-0.0174	1.32	0.339	0.276
**2B**	0.0517	0.1047	-0.0101	1.28	0.297	0.244
**2C**	0.0462	0.0914	-0.0077	1.25	0.284	0.222
**2D**	0.0394	0.0767	-0.0051	1.21	0.266	0.193
**3A** [a]	0.0623; 0.0632; 0.0629	0.1404; 0.1442; 0.1434	-0.0149; -0.0152; -0.0150	1.30; 1.30; 1.29	0.302; 0.305; 0.313	0.252; 0.257; 0.255
**3B** [a]	0.0471; 0.0476; 0.0465	0.0967; 0.0986; 0.0962	-0.0082; -0.0083; -0.0078	1.25; 1.25; 1.25	0.258; 0.261; 0.265	0.218; 0.221; 0.217
**3C** [a]	0.0417; 0.0422; 0.0408	0.0840; 0.0859; 0.0829	-0.0060; -0.0062; -0.0056	1.22; 1.22; 1.21	0.245; 0.248; 0.249	0.196; 0.199; 0.194
**3D** [a]	0.0345; 0.0349; 0.0322	0.0693; 0.0705; 0.0648	-0.0035; -0.0036; -0.0027	1.17; 1.17; 1.14	0.223; 0.226; 0.219	0.166; 0.169; 0.159
**4A**	0.0638	0.1465	-0.0153	1.30	0.320	0.262
**4B**	0.0478	0.1001	-0.0083	1.25	0.273	0.225
**4C**	0.0419	0.0861	-0.0059	1.22	0.257	0.201
**4D**	0.0330	0.0671	-0.0029	1.15	0.225	0.165
**5A**	0.0571	0.1308	-0.0122	1.27	0.273	0.228
**5B**	0.0403	0.0846	-0.0055	1.21	0.219	0.184
**5C**	0.0355	0.0732	-0.0038	1.17	0.208	0.167
**5D**	0.0273	0.0569	-0.0014	1.09	0.177	0.134

[a] Results are given for N→Sn involving the N atom at position 1, 2 or 4, respectively.

Table S9. QTAIM and NPA charges (in a.u.) of the atoms forming the N→Sn bonds in the 1:1 conventional coordination complexes of azines and SnX_4.

Complex	QTAIM		NPA	
	N	Sn	N	Sn
**1A**	-1.140	2.536	-0.535	2.465
**1B**	-1.141	1.923	-0.497	1.462
**1C**	-1.141	1.603	-0.493	1.160
**1D**	-1.142	1.210	-0.490	0.730
**2A**	-1.138	2.537	-0.480	2.467
**2B**	-1.138	1.921	-0.443	1.462
**2C**	-1.137	1.600	-0.437	1.160
**2D**	-1.137	1.206	-0.434	0.729
**3A** [a]	-0.636;<br>-0.641;<br>-1.127	2.542;<br>2.542;<br>2.539	-0.267;<br>-0.315;<br>-0.497	2.468;<br>2.467;<br>2.469
**3B** [a]	-0.631;<br>-0.637;<br>-1.125	1.921;<br>1.923;<br>1.916	-0.231;<br>-0.281;<br>-0.458	1.462;<br>1.462;<br>1.454
**3C** [a]	-0.627;<br>-0.634;<br>-1.123	1.597;<br>1.599;<br>1.592	-0.227;<br>-0.277;<br>-0.452	1.159;<br>1.160;<br>1.147
**3D** [a]	-0.624;<br>-0.632;<br>-1.120	1.198;<br>1.201;<br>1.188	-0.223;<br>-0.274;<br>-0.444	0.728;<br>0.729;<br>0.708
**4A**	-1.130	2.540	-0.546	2.470
**4B**	-1.131	1.921	-0.510	1.458
**4C**	-1.131	1.597	-0.505	1.152
**4D**	-1.129	1.194	-0.498	0.713
**5A**	-0.607	2.543	-0.271	2.470
**5B**	-0.599	1.914	-0.238	1.455
**5C**	-0.577	1.581	-0.229	1.137
**5D**	-0.571	1.174	-0.224	0.697

[a] Results are given for N→Sn involving the N atom at positions 1, 2 or 4, respectively.

Table S10. LMOEDA components (in kJ mol^{-1}) of the pairwise interaction between the molecular fragments of azine and SnX_4 in the 1:1 conventional coordination complexes of azines and SnX_4. The percentage share of each attractive component with respect to the pairwise attraction is given in parentheses.

Complex	E_{int}	ε_{elst} (%ε_{elst})	ε_{pol} (%ε_{pol})	ε_{disp} (%ε_{disp})	$\varepsilon_{exch\text{-}rep}$
**1A**	-171.4	-238.2 (51.0)	-181.3 (38.8)	-47.8 (10.2)	295.8
**1B**	-112.6	-194.6 (48.3)	-154.8 (38.4)	-53.7 (13.3)	290.3
**1C**	-93.3	-179.1 (47.4)	-142.8 (37.8)	-56.0 (14.8)	284.5
**1D**	-70.1	-157.8 (46.3)	-124.1 (36.4)	-59.2 (17.4)	271.0
**2A**	-165.7	-229.6 (50.6)	-176.2 (38.9)	-47.7 (10.5)	287.8
**2B**	-109.0	-188.0 (47.9)	-150.3 (38.3)	-53.8 (13.7)	283.2
**2C**	-89.8	-171.7 (47.1)	-137.0 (37.6)	-55.9 (15.3)	274.8
**2D**	-67.6	-151.7 (46.0)	-119.2 (36.1)	-59.1 (17.9)	262.3
**3A** [a]	-149.0	-200.3 (50.4)	-154.2 (38.8)	-43.0 (10.8)	248.4
**3B** [a]	-96.0	-160.8 (47.7)	-128.2 (38.1)	-47.9 (14.2)	241.0
**3C** [a]	-78.6	-145.3 (46.8)	-115.9 (37.3)	-49.4 (15.9)	231.9
**3D** [a]	-58.4	-125.5 (45.5)	-98.1 (35.6)	-51.8 (18.8)	217.1
**4A**	-150.5	-215.2 (50.6)	-163.9 (38.6)	-45.9 (10.8)	274.6
**4B**	-92.8	-171.8 (48.1)	-133.8 (37.5)	-51.5 (14.4)	264.3
**4C**	-74.0	-154.5 (47.3)	-118.9 (36.4)	-53.4 (16.3)	252.8
**4D**	-51.1	-126.8 (46.0)	-93.8 (34.0)	-55.3 (20.0)	224.7
**5A**	-123.3	-171.3 (49.6)	-133.5 (38.6)	-40.9 (11.8)	222.3
**5B**	-72.1	-129.5 (47.1)	-100.6 (36.6)	-44.9 (16.3)	202.9
**5C**	-54.8	-117.4 (46.0)	-89.7 (35.1)	-48.2 (18.9)	200.6
**5D**	-37.6	-96.2 (44.5)	-69.8 (32.3)	-49.9 (23.1)	178.2

[a] Results are given for N→Sn involving the N atom at position 1.

Section S6. Additional tables and figures

Table S11. Results of the search through the CSD for structures showing an azine moiety coordinating two tin(IV) atoms.

Structures showing the coordination of two Sn(IV) sites with the nitrogen sites of a pyrimidine moiety	Structures showing the coordination of two Sn(IV) sites with the nitrogen sites of a pyrazine moiety	Structures showing the coordination of two Sn(IV) sites with the nitrogen sites of a 1,3,5-triazine moiety
QEHDAJ	CORMIF10	YELLUX
QEHDEN	FARVID01	YELMAE
QEHDIR	FARVOJ	YELMEI
FUCBOU10 [a]	FIHMOY	YELMIM
ZOFFIJ [a]	FIHMUE	

[a] A single pyrimidine ring coordinated by two Sn(IV) atoms belongs to a 2,2'-bipyrimidine unit.

Table S12. Geometrical (in pm) and energetic parameters (in kJ mol^{-1}) for the inverse coordination complexes of two SnX_4 molecules attached at various positions to an azine center.

Complex	*d*	$E_{complex}$	E_{int} [a]	E_{def}		ΔZPVE
				azine	$2SnX_4$	
1,3-coordination						
**1A**$_2$	234.5	-191.2	-268.2 (-266.6)	6.6	60.3	10.0
**1B**$_2$	250.9	-98.6	-177.1 (-176.4)	3.9	66.1	8.5
**1C**$_2$	258.5	-87.3	-153.5 (-153.9)	2.7	56.7	6.9
**1D**$_2$	271.0	-79.3	-131.4 (-127.7)	1.8	44.7	5.7
**3A**$_2$	238.6; 238.7 [b]	-162.6	-227.3 (-226.1)	6.2	48.8	9.7
**3B**$_2$	255.1; 258.1 [b]	-79.2	-142.9 (-142.4)	3.7	52.3	7.8
**3C**$_2$	260.9; 268.5 [b]	-70.9	-123.9 (-124.3)	2.9	43.6	6.5
**3D**$_2$	262.0; 315.5 [b]	-70.3	-107.0 (-104.1)	2.2	29.1	5.4
**4A**$_2$	237.4	-167.1	-232.4 (-231.2)	6.5	49.6	9.2
**4B**$_2$	255.4	-80.1	-144.9 (-144.5)	3.6	54.0	7.2
**4C**$_2$	264.8	-71.8	-125.1 (-125.5)	2.4	44.7	6.1
**4D**$_2$	278.2	-62.5	-104.2 (-101.1)	1.8	35.1	4.9
**5A**$_2$	243.4	-132.6	-186.8 (-185.8)	6.3	38.9	9.1
**5B**$_2$	265.1	-58.9	-108.4 (-108.0)	3.6	38.8	7.1
**5C**$_2$	271.8	-55.7	-96.1 (-96.5)	2.7	31.6	6.2
**5D**$_2$	303.7	-51.0	-70.4 (-69.2)	1.1	14.1	4.1
1,4-coordination						
**2A**$_2$	235.0	-190.6	-265.0 (-263.5)	4.2	59.6	10.7
**2B**$_2$	251.2	-97.0	-173.6 (-172.8)	2.3	65.7	8.5
**2C**$_2$	257.9	-83.7	-152.4 (-152.7)	1.9	59.5	7.3
**2D**$_2$	269.1	-71.4	-127.5 (-123.2)	1.4	48.7	5.9
**3A**$_2$	238.2; 238.6 [c]	-163.9	-228.9 (-227.5)	4.8	50.2	10.0
**3B**$_2$	255.3; 257.0 [c]	-79.0	-143.9 (-143.3)	2.9	54.0	8.0
**3C**$_2$	261.8; 266.0 [c]	-68.8	-124.7 (-125.1)	2.4	46.7	6.8
**3D**$_2$	267.3; 309.9 [c]	-61.6	-98.0 (-95.2)	1.7	29.3	5.4
**5A**$_2$	243.0	-134.6	-189.4 (-188.4)	5.2	40.6	9.0
**5B**$_2$	264.4	-58.8	-109.0 (-108.6)	3.0	40.0	7.2
**5C**$_2$	271.7	-52.1	-94.1 (-94.5)	2.4	33.2	6.4
**5D**$_2$	297.8	-50.2	-72.5 (-71.1)	1.2	16.4	4.8

[a] Interaction energies including both scalar and spin-orbit relativistic corrections are given in parentheses. [b] Results are given for N→Sn involving the N atoms at positions 2 and 4, respectively. [c] Results are given for N→Sn involving the N atoms at positions 1 and 4, respectively.

Table S13. Many-body analysis of the total interaction energy (in kJ mol^{-1}) calculated at the CCSD(T)/CBS level for the inverse coordination complexes of two SnX_4 molecules attached at various positions to an azine center.

Complex	E_{int}	$\varepsilon_{int}(azine,SnX_4)$	$\varepsilon_{int}(SnX_4,SnX_4')$	$\varepsilon_{int}(azine,SnX_4,SnX_4')$
1,3-coordination				
**1A**$_2$	-295.1	-160.8	5.0	21.5
**1B**$_2$	-191.8	-104.3	-1.3	18.2
**1C**$_2$	-155.5	-81.6	-9.2	16.8
**1D**$_2$	-120.2	-60.2	-8.2	8.3
**3A**$_2$	-248.5	-141.4; -133.1 [a]	3.9	22.1
**3B**$_2$	-153.6	-88.1; -77.3 [a]	-1.5	13.2
**3C**$_2$	-121.1	-68.7; -59.6 [a]	-5.1	12.3
**3D**$_2$	-92.6	-57.4; -27.8 [a]	-8.4	1.0
**4A**$_2$	-252.6	-139.5	3.5	23.0
**4B**$_2$	-155.0	-83.2	-1.4	13.0
**4C**$_2$	-123.3	-65.0	-6.1	12.8
**4D**$_2$	-93.3	-45.9	-4.4	2.9
**5A**$_2$	-202.5	-113.2	3.4	20.6
**5B**$_2$	-116.8	-63.5	-1.6	11.8
**5C**$_2$	-88.8	-46.4	-6.3	10.4
**5D**$_2$	-63.1	-32.7	-4.4	6.7
1,4-coordination				
**2A**$_2$	-288.2	-158.3	3.4	25.0
**2B**$_2$	-188.4	-101.6	0.5	14.4
**2C**$_2$	-152.3	-80.7	-0.1	9.1
**2D**$_2$	-118.5	-61.3	-1.7	5.9
**3A**$_2$	-250.6	-138.8; -133.5 [b]	3.1	18.6
**3B**$_2$	-154.1	-87.2; -78.7 [b]	0.1	11.7
**3C**$_2$	-124.0	-72.8; -62.6 [b]	-0.1	11.5
**3D**$_2$	-89.8	-58.6; -31.3 [b]	-1.8	1.9
**5A**$_2$	-204.8	-113.2	2.6	19.0
**5B**$_2$	-116.5	-63.2	0.5	9.4
**5C**$_2$	-87.2	-47.7	-0.6	8.9
**5D**$_2$	-60.2	-29.5	-1.4	0.4

[a] Results are given for SnX_4 coordinated to the N atoms at positions 2 and 4, respectively. [b] Results are given for SnX_4 coordinated to the N atoms at positions 1 and 4, respectively.

Table S14. Differences in the geometrical (Δd, in pm), energetic ($\Delta\varepsilon_{int}(azine,SnX_4)$, in kJ mol^{-1}) and electronic (ΔQ_{azine}, in a.u.) parameters of the 1:2 inverse coordination complexes relative to the corresponding parameters in the 1:1 conventional coordination complexes.

Complex	Δd [a]	$\Delta\varepsilon_{int}(azine,SnX_4)$ [b]	ΔQ_{azine} [c]	
			QTAIM	NPA
1,3-coordination				
**1A**$_2$	4.5	16.7	0.116	0.111
**1B**$_2$	7.1	14.0	0.054	0.085
**1C**$_2$	9.2	13.8	0.018	0.055
**1D**$_2$	13.1	15.2	0.006	0.026
**3A**$_2$ [d]	5.3; 5.3	12.7; 12.6	0.102	0.103
**3B**$_2$ [d]	6.9; 8.9	10.7; 15.6	0.049	0.081
**3C**$_2$ [d]	6.3; 12.3	10.4; 11.5	0.029	0.062
**3D**$_2$ [d]	0.7; 46.5	3.7; 32.6	0.032	0.078
**4A**$_2$	4.8	14.3	0.103	0.100
**4B**$_2$	7.7	12.4	0.036	0.069
**4C**$_2$	10.2	9.8	0.007	0.040
**4D**$_2$	10.8	8.3	-0.020	0.022
**5A**$_2$	5.6	10.4	0.081	0.081
**5B**$_2$	9.1	9.3	0.020	0.044
**5C**$_2$	8.6	8.0	0.007	0.012
**5D**$_2$	26.2	4.4	-0.034	-0.010
1,4-coordination				
**2A**$_2$	4.3	14.6	0.124	0.121
**2B**$_2$	6.6	13.4	0.060	0.093
**2C**$_2$	7.4	9.8	0.036	0.076
**2D**$_2$	10.0	10.2	0.002	0.048
**3A**$_2$ [e]	4.0; 5.2	13.5; 12.2	0.109	0.106
**3B**$_2$ [e]	6.3; 7.8	10.0; 14.2	0.050	0.081
**3C**$_2$ [e]	6.4; 9.8	5.0; 8.5	0.032	0.066
**3D**$_2$ [e]	1.8; 40.9	1.7; 29.1	0.040	0.082
**5A**$_2$	5.2	10.4	0.088	0.088
**5B**$_2$	8.4	9.6	0.024	0.052
**5C**$_2$	8.5	6.7	0.002	0.030
**5D**$_2$	20.3	7.6	-0.041	-0.008

[a] Positive values mean longer N→Sn bonds in the 1:2 complexes. [b] Positive values mean energetically less favorable interaction energies for the 1:2 complexes. [c] Positive values mean more positive charges acquired by the azine fragment in the 1:2 complexes. [d] Results are given for SnX_4 coordinated to the N atoms at positions 2 and 4, respectively. [e] Results are given for SnX_4 coordinated to the N atoms at positions 1 and 4, respectively.

Table S15. LMOEDA components (in kJ mol^{-1}) of the three-body contribution to E_{int} for the inverse coordination complexes of two SnX_4 molecules attached at various positions to an azine center.

Complex	$\varepsilon_{int}(azine, SnX_4, SnX_4')$	ε_{pol}	ε_{disp}	$\varepsilon_{exch\text{-}rep}$
1,3-coordination				
**1A**$_2$	26.4	26.0	2.5	-2.1
**1B**$_2$	17.3	18.1	1.6	-2.4
**1C**$_2$	13.8	15.8	1.1	-3.1
**1D**$_2$	7.2	10.4	1.1	-4.3
**3A**$_2$	24.2	23.7	2.3	-1.9
**3B**$_2$	14.8	14.9	1.3	-1.5
**3C**$_2$	9.8	11.6	1.0	-2.7
**3D**$_2$	0.2	3.4	0.8	-4.0
**4A**$_2$	23.9	23.5	2.2	-1.7
**4B**$_2$	13.9	14.2	1.2	-1.6
**4C**$_2$	9.2	11.1	0.9	-2.8
**4D**$_2$	3.5	5.6	0.8	-2.9
**5A**$_2$	21.7	21.2	2.1	-1.7
**5B**$_2$	11.1	11.5	1.0	-1.4
**5C**$_2$	6.1	7.9	0.8	-2.6
**5D**$_2$	1.2	2.6	0.6	-2.0
1,4-coordination				
**2A**$_2$	21.9	22.0	1.2	-1.3
**2B**$_2$	15.0	15.5	0.3	-0.8
**2C**$_2$	11.9	12.4	0.0	-0.5
**2D**$_2$	8.2	8.7	-0.2	-0.3
**3A**$_2$	20.4	20.4	1.2	-1.3
**3B**$_2$	12.5	12.9	0.3	-0.7
**3C**$_2$	9.2	9.6	0.0	-0.4
**3D**$_2$	2.8	3.1	-0.2	-0.1
**5A**$_2$	18.9	18.7	1.1	-1.0
**5B**$_2$	10.0	10.3	0.2	-0.5
**5C**$_2$	6.3	6.6	0.0	-0.3
**5D**$_2$	1.8	2.0	-0.2	0.0

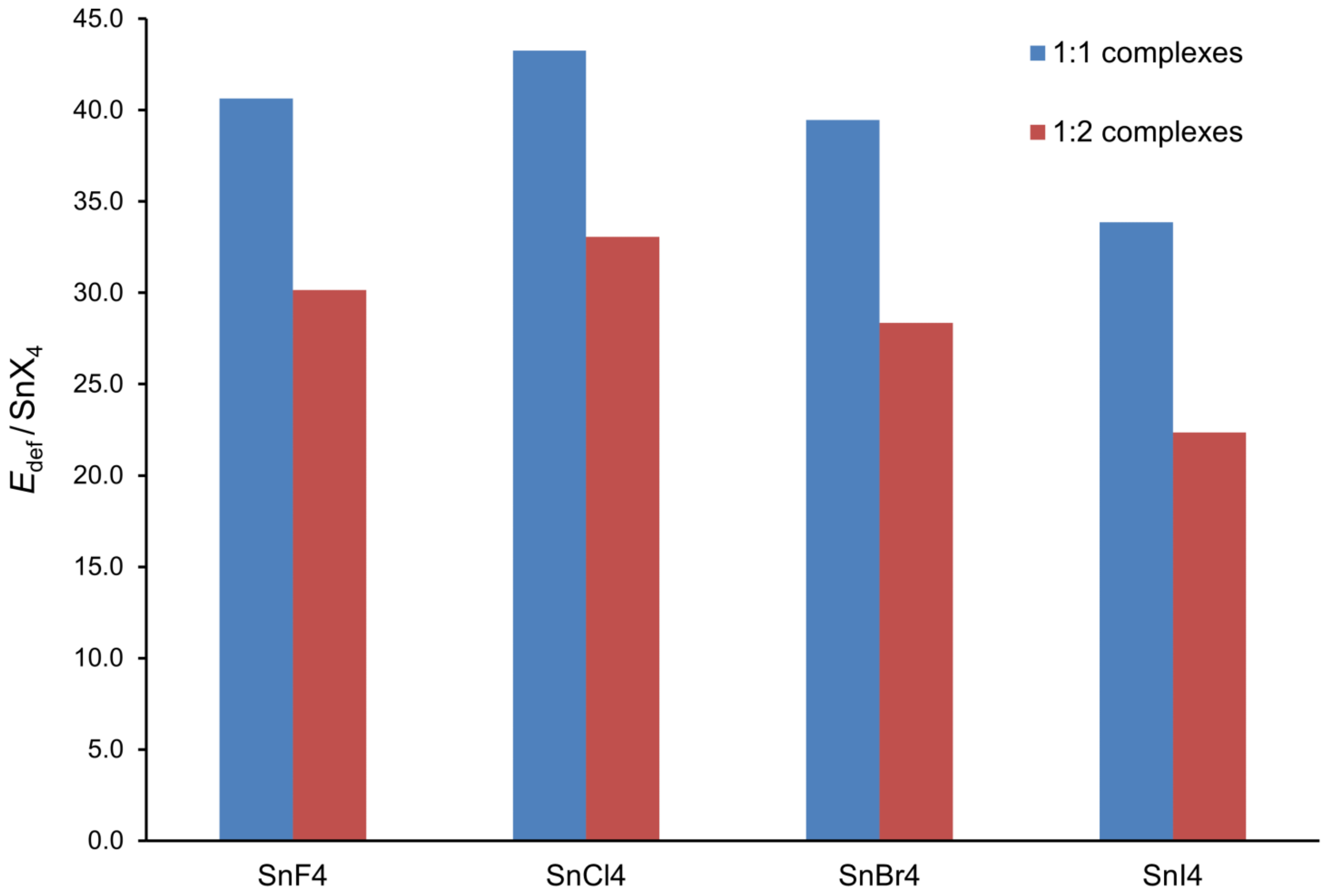

Figure S4. Variations in the deformation energy per SnX_4 molecule (E_{def} / SnX_4, in kJ mol^{-1}) for the formation of the 1:1 and 1:2 complexes of **1** with SnX_4.

References

(1) Becke, A. D. Density-Functional Exchange-Energy Approximation with Correct Asymptotic Behavior. *Phys. Rev. A* **1988**, *38*, 3098–3100.

(2) Perdew, J. P. Density-Functional Approximation for the Correlation Energy of the Inhomogeneous Electron Gas. *Phys. Rev. B* **1986**, *33*, 8822–8824.

(3) Grimme, S. Semiempirical GGA-Type Density Functional Constructed with a Long-Range Dispersion Correction. *J. Comput. Chem.* **2006**, *27*, 1787–1799.

(4) Dunning, T. H., Jr. Gaussian Basis Sets for Use in Correlated Molecular Calculations. I. The Atoms Boron through Neon and Hydrogen. *J. Chem. Phys.* **1989**, *90*, 1007–1023.

(5) Metz, B.; Stoll, H.; Dolg, M. Small-Core Multiconfiguration-Dirac–Hartree–Fock-Adjusted Pseudopotentials for Post-d Main Group Elements: Application to PbH and PbO. *J. Chem. Phys.* **2000**, *113*, 2563–2569.

(6) Peterson, K. A. Systematically Convergent Basis Sets with Relativistic Pseudopotentials. I. Correlation Consistent Basis Sets for the Post-d Group 13–15 Elements. *J. Chem. Phys.* **2003**, *119*, 11099–11112.

(7) Eichkorn, K.; Treutler, O.; Öhm, H.; Häser, M.; Ahlrichs, R. Auxiliary Basis Sets to Approximate Coulomb Potentials. *Chem. Phys. Lett.* **1995**, *240*, 283–290.

(8) Eichkorn, K.; Weigend, F.; Treutler, O.; Ahlrichs, R. Auxiliary Basis Sets for Main Row Atoms and Transition Metals and Their Use to Approximate Coulomb Potentials. *Theor. Chem. Acc.* **1997**, *97*, 119–124.

(9) Weigend, F. Accurate Coulomb-Fitting Basis Sets for H to Rn. *Phys. Chem. Chem. Phys.* **2006**, *8*, 1057–1065.

(10) Iliaš, M.; Saue, T. An Infinite-Order Two-Component Relativistic Hamiltonian by a Simple One-Step Transformation. *J. Chem. Phys.* **2007**, *126*, No. 064102.

(11) Peng, D.; Middendorf, N.; Weigend, F.; Reiher, M. An Efficient Implementation of Two-Component Relativistic Exact-Decoupling Methods for Large Molecules. *J. Chem. Phys.* **2013**, *138*, No. 184105.

(12) De Jong, W. A.; Harrison, R. J.; Dixon, D. A. Parallel Douglas–Kroll Energy and Gradients in NWChem: Estimating Scalar Relativistic Effects Using Douglas–Kroll Contracted Basis Sets. *J. Chem. Phys.* **2001**, *114*, 48–53.

(13) Bross, D. H.; Peterson, K. A. Correlation Consistent, Douglas–Kroll–Hess Relativistic Basis Sets for the 5p and 6p Elements. *Theor. Chem. Acc.* **2014**, *133*, No. 1434.

(14) Gauss, J. Coupled-Cluster Theory. In *Encyclopedia of Computational Chemistry*; Schleyer, P. v. R., Allinger, N. L., T. Clark, Gasteiger, J., Kollman, P. A., Schaefer III, H. F., Schreiner, P. R., Eds.; Wiley: Chichester, UK, 1998; pp 615–636.

(15) Schmitz, G.; Hättig, C.; Tew, D. P. Explicitly Correlated PNO-MP2 and PNO-CCSD and Their Application to the S66 Set and Large Molecular Systems. *Phys. Chem. Chem. Phys.* **2014**, *16*, 22167–22178.

(16) Schmitz, G.; Hättig, C. Perturbative Triples Correction for Local Pair Natural Orbital Based Explicitly Correlated CCSD(F12*) Using Laplace Transformation Techniques. *J. Chem. Phys.* **2016**, *145*, No. 234107.

(17) Weigend, F.; Köhn, A.; Hättig, C. Efficient Use of the Correlation Consistent Basis Sets in Resolution of the Identity MP2 Calculations. *J. Chem. Phys.* **2002**, *116*, 3175–3183.

(18) Hättig, C.; Schmitz, G.; Koßmann, J. Auxiliary Basis Sets for Density-Fitted Correlated Wavefunction Calculations: Weighted Core-Valence and ECP Basis Sets for Post-d Elements. *Phys. Chem. Chem. Phys.* **2012**, *14*, 6549–6555.

(19) Feller, D. The Use of Systematic Sequences of Wave Functions for Estimating the Complete Basis Set, Full Configuration Interaction Limit in Water. *J. Chem. Phys.* **1993**, *98*, 7059–7071.

(20) Pansini, F. N. N.; Neto, A. C.; Varandas, A. J. C. Extrapolation of Hartree–Fock and Multiconfiguration Self-Consistent-Field Energies to the Complete Basis Set Limit. *Theor. Chem. Acc.* **2016**, *135*, No. 261.

(21) Halkier, A.; Helgaker, T.; Jørgensen, P.; Klopper, W.; Koch, H.; Olsen, J.; Wilson, A. K. Basis-Set Convergence in Correlated Calculations on Ne, N_2, and H_2O. *Chem. Phys. Lett.* **1998**, *286*, 243–252.

(22) Varandas, A. J. C.; Pansini, F. N. N. Narrowing the Error in Electron Correlation Calculations by Basis Set Re-Hierarchization and Use of the Unified Singlet and Triplet Electron-Pair Extrapolation Scheme: Application to a Test Set of 106 Systems. *J. Chem. Phys.* **2014**, *141*, No. 224113.

(23) Boys, S. F.; Bernardi, F. The Calculation of Small Molecular Interactions by the Differences of Separate Total Energies. Some Procedures with Reduced Errors. *Mol. Phys.* **1970**, *19*, 553–566.

(24) Su, P.; Li, H. Energy Decomposition Analysis of Covalent Bonds and Intermolecular Interactions. *J. Chem. Phys.* **2009**, *131*, No. 014102.

(25) Furness, J. W.; Kaplan, A. D.; Ning, J.; Perdew, J. P.; Sun, J. Accurate and Numerically Efficient r^2SCAN Meta-Generalized Gradient Approximation. *J. Phys. Chem. Lett.* **2020**, *11*, 8208–8215.

(26) Caldeweyher, E.; Bannwarth, C.; Grimme, S. Extension of the D3 Dispersion Coefficient Model. *J. Chem. Phys.* **2017**, *147*, No. 034112.

(27) Caldeweyher, E.; Ehlert, S.; Hansen, A.; Neugebauer, H.; Spicher, S.; Bannwarth, C.; Grimme, S. A Generally Applicable Atomic-Charge Dependent London Dispersion Correction. *J. Chem. Phys.* **2019**, *150*, No. 154122.

(28) Bader, R. F. W. *Atoms in Molecules: A Quantum Theory*; Clarendon: Oxford, UK, 1990.

(29) Keith, T. A. *AIMAll 19.10.12*; TK Gristmill Software: Overland Park, KS, USA, 2019.

(30) Weinhold, F.; Landis, C. R. *Valency and Bonding: A Natural Bond Orbital Donor–Acceptor Perspective*; Cambridge University Press: New York, USA, 2005.

(31) Reed, A. E.; Weinstock, R. B.; Weinhold, F. Natural Population Analysis. *J. Chem. Phys.* **1985**, *83*, 735–746.

(32) Ahlrichs, R.; Armbruster, M. K.; Bachorz, R. A.; Bahmann, H.; Baldes, A.; Bär, M.; Baron, H.; Bauernschmitt, R.; Bischof, F. A.; Böcker, S.; Burow, A. M.; Chen, G. P.; Crawford, N.; Deglmann, P.; Della Sala, F.; Diedenhofen, M.; Ehlert, S.; Ehrig, M.; Eichkorn, K.; Elliott, S.; Franzke, Y. J.; Friese, D.; Furche, F.; Balasubramani, S. G.; Gimon, T.; Glöß, A.; Graf, N.; Grajciar, L.; Grotjahn, R. G.; Haase, F.; Häser, M.; Hättig, C.; Hellweg, A.; Helmich, B.; Höfener, S.; Holzer, C.; Horn, H.; Huber, C.; Hujo, W.; Huniar, U.; Kattannek, M.; Kehry, M.; Klawohn, S.; Klopper, W.; Köhn, A.; Kölmel, C.; Kollwitz, M.; Krause, K.; Kühn, M.; Łazarski, R.; Maier, T. M.; Mack, F.; May, K.; Middendorf, N.; Nava, P.; Ochsenfeld, C.; Öhm, H.; Pabst, M.; Parker, S. M.; Patzelt, H.; Pausch, A.; Pollak, P.;

Rappoport, D.; Reiter, K.; Roy, S.; Rubner, O.; Schäfer, A.; Schmitz, G.; Schneider, U.; Schwabe, T.; Sierka, M.; Tew, D. P.; Treutler, O.; Unterreiner, B.; von Arnim, M.; Voora, V. K.; Weigend, F.; Weis, P.; Weiss, H.; Winter, N.; Yu, J. M. *TURBOMOLE 7.7*; TURBOMOLE GmbH: Karlsruhe, Germany, 2022.

(33) Frisch, M. J.; Trucks, G. W.; Schlegel, H. B.; Scuseria, G. E.; Robb, M. A.; Cheeseman, J. R.; Scalmani, G.; Barone, V.; Mennucci, B.; Petersson, G. A.; Nakatsuji, H.; Caricato, M.; Li, X.; Hratchian, H. P.; Izmaylov, A. F.; Bloino, J.; Zheng, G.; Sonnenberg, J. L.; Hada, M.; Ehara, M.; Toyota, K.; Fukuda, R.; Hasegawa, J.; Ishida, M.; Nakajima, T.; Honda, Y.; Kitao, O.; Nakai, H.; Vreven, T.; Montgomery Jr., J. A.; Peralta, J. E.; Ogliaro, F.; Bearpark, M.; Heyd, J. J.; Brothers, E.; Kudin, K. N.; Staroverov, V. N.; Keith, T.; Kobayashi, R.; Normand, J.; Raghavachari, K.; Rendell, A.; Burant, J. C.; Iyengar, S. S.; Tomasi, J.; Cossi, M.; Rega, N.; Millam, J. M.; Klene, M.; Knox, J. E.; Cross, J. B.; Bakken, V.; Adamo, C.; Jaramillo, J.; Gomperts, R.; Stratmann, R. E.; Yazyev, O.; Austin, A. J.; Cammi, R.; Pomelli, C.; Ochterski, J. W.; Martin, R. L.; Morokuma, K.; Zakrzewski, V. G.; Voth, G. A.; Salvador, P.; Dannenberg, J. J.; Dapprich, S.; Daniels, A. D.; Farkas, O.; Foresman, J. B.; Ortiz, J. V.; Cioslowski, J.; Fox, D. J. *Gaussian 09 D.01*; Gaussian, Inc.: Wallingford, CT, USA, 2013.

(34) Glendening, E. D.; Badenhoop, J. K.; Reed, A. E.; Carpenter, J. E.; Bohmann, J. A.; Morales, C. M.; Landis, C. R.; Weinhold, F. *NBO 6.0*; Theoretical Chemistry Institute, University of Wisconsin: Madison, WI, USA, 2013.

(35) Lu, T.; Chen, F. Multiwfn: A Multifunctional Wavefunction Analyzer. *J. Comput. Chem.* **2012**, *33*, 580–592.

(36) Groom, C. R.; Bruno, I. J.; Lightfoot, M. P.; Ward, S. C. The Cambridge Structural Database. *Acta Cryst. B* **2016**, *72*, 171–179.

(37) Bruno, I. J.; Cole, J. C.; Edgington, P. R.; Kessler, M.; Macrae, C. F.; McCabe, P.; Pearson, J.; Taylor, R. New Software for Searching the Cambridge Structural Database and Visualizing Crystal Structures. *Acta Cryst. B* **2002**, *58*, 389–397.

(38) Matczak, P.; Łukomska, M. Assessment of Various Density Functionals for Intermolecular N→Sn Interactions: The Test Case of Trimethyltin Cyanide Dimer. *Comput. Theoret. Chem.* **2014**, *1036*, 31–43.

(39) Matczak, P.; Wojtulewski, S. Performance of Møller-Plesset Second-Order Perturbation Theory and Density Functional Theory in Predicting the Interaction between Stannylenes and Aromatic Molecules. *J. Mol. Model.* **2015**, *21*, No. 41.

(40) Matczak, P. N→Sn Coordination in the Complexes of Tin Halides with Pyridine: A Comparison between Sn(II) and Sn(IV). *Appl. Organometal. Chem.* **2019**, *33*, No. e4811.

(41) Bankiewicz, B.; Kupfer, S.; Matczak, P. Tuning the Metal–Ligand Bond in the σ-Complexes of Stannylenes and Azabenzenes. *J. Comput. Chem.* **2021**, *42*, 2103–2115.

(42) Matczak, P. Interplay between Different Metal–Ligand Binding Modes in Tin(II) Complexes with Pyridine. *Comput. Theoret. Chem.* **2023**, *1226*, No. 114192.

(43) Matczak, P. Theoretical Insight into the Interaction between SnX_2 (X=H, F, Cl, Br, I) and Benzene. *J. Mol. Model.* **2016**, *22*, 208.

(44) Grabowski, S. J. Tetrel Bonds, Penta- and Hexa-coordinated Tin and Lead Centres. *Appl. Organomet. Chem.* **2017**, *31*, No. e3727.

(45) Michalczyk, M.; Zierkiewicz, W.; Wysokiński, R.; Scheiner, S. Hexacoordinated Tetrel-Bonded Complexes between TF_4 (T=Si, Ge, Sn, Pb) and NCH: Competition between σ- and π-Holes. *ChemPhysChem* **2019**, *20*, 959–966.

(46) Scheiner, S. Origins and Properties of the Tetrel Bond. *Phys. Chem. Chem. Phys.* **2021**, *23*, 5702–5717.

(47) Řezáč, J.; Riley, K. E.; Hobza, P. Benchmark Calculations of Noncovalent Interactions of Halogenated Molecules. *J. Chem. Theory Comput.* **2012**, *8*, 4285−4292.

(48) Pašteka, L. F.; Rajský, T.; Urban, M. Toward Understanding the Bonding Character in Complexes of Coinage Metals with Lone-Pair Ligands. CCSD(T) and DFT Computations. *J. Phys. Chem. A* **2013**, *117*, 4472–4485.

(49) Steinmetz, M.; Grimme, S. Benchmark Study of the Performance of Density Functional Theory for Bond Activations with (Ni,Pd)-Based Transition-Metal Catalysts. *ChemistryOpen* **2013**, *2*, 115–124.

(50) Zhao, Y.; Truhlar, D. G. The M06 Suite of Density Functionals for Main Group Thermochemistry, Thermochemical Kinetics, Noncovalent Interactions, Excited States, and Transition Elements: Two New Functionals and Systematic Testing of Four M06-Class Functionals and 12 Other Functionals. *Theor. Chem. Acc.* **2008**, *120*, 215–241.

(51) Bankiewicz, B.; Matczak, P. Controlling the Preferred Nitrogen Site in 1,2,3-Triazine to Bind with Stannylenes. *Polyhedron* **2022**, *225*, No. 116056.

(52) Ehlert, S.; Huniar, U.; Ning, J.; Furness, J. W.; Sun, J.; Kaplan, A. D.; Perdew, J. P.; Brandenburg, J. G. r^2SCAN-D4: Dispersion Corrected Meta-Generalized Gradient Approximation for General Chemical Applications. *J. Chem. Phys.* **2021**, *154*, No. 061101.

(53) Chattaraj, P. K.; Maiti, B.; Sarkar, U. Philicity: A Unified Treatment of Chemical Reactivity and Selectivity. *J. Phys. Chem. A* **2003**, *107*, 4973–4975.

(54) Domingo, L. R.; Pérez, P.; Sáez, J. A. Understanding the Local Reactivity in Polar Organic Reactions through Electrophilic and Nucleophilic Parr Functions. *RSC Adv.* **2013**, *3*, 1486–1494.

(55) Parr, R. G.; Yang, W. *Density-Functional Theory of Atoms and Molecules*; Oxford University Press: New York, USA, 1989.

(56) Bader, R. F. W. A Bond Path: A Universal Indicator of Bonded Interactions. *J. Phys. Chem. A* **1998**, *102*, 7314–7323.

(57) Bader, R. F. W.; Essén, H. The Characterization of Atomic Interactions. *J. Chem. Phys.* **1984**, *80*, 1943–1960.

(58) Espinosa, E.; Alkorta, I.; Elguero, J.; Molins, E. From Weak to Strong Interactions: A Comprehensive Analysis of the Topological and Energetic Properties of the Electron Density Distribution Involving X–H⋯F–Y Systems. *J. Chem. Phys.* **2002**, *117*, 5529–5542.

(59) Sánchez-Coronilla, A.; Sánchez-Márquez, J.; Zorrilla, D.; Martın, E. I.; de los Santos, D. M.; Navas, J.; Fernández-Lorenzo, C.; Alcántara, R.; Martın-Calleja, J. Convergent Study of Ru–Ligand Interactions through QTAIM, ELF, NBO Molecular Descriptors and TDDFT Analysis of Organometallic Dyes. *Mol. Phys.* **2014**, *112*, 2063–2077.

(60) Hosseinnejad, T.; Kazemi, T. Quantum Chemical Investigation on Complexation of Palladium with Iminopyridyl Ligands: Structural, Thermochemical, and Electronic Aspects. *Mol. Cryst. Liq. Cryst.* **2016**, *637*, 53–64.

(61) Alexiou, A. D. P.; Decandio, C. C.; da N. Almeida, S.; Ferreira, M. J. P.; Romoff, P.; Rocha, R. C. Metal-Ligand Coordination and Antiradical Activity of a Trichromium(III) Complex with the Flavonoid Naringenin. *J. Coord. Chem.* **2017**, *70*, 2148–2160.

(62) Grenon, N.; Baumgartner, T. Exploration of Hypervalent Lewis Acid/Base Interactions in 2-(2′-Thiazolyl)-3-Thienylphosphanes. *Inorg. Chem.* **2018**, *57*, 1630−1644.

(63) Fradera, X.; Austen, M. A.; Bader, R. F. W. The Lewis Model and Beyond. *J. Phys. Chem. A* **1999**, *103*, 304–314.

(64) Wiberg, K. B. Application of the Pople-Santry-Segal CNDO Method to the Cyclopropylcarbinyl and Cyclobutyl Cation and to Bicyclobutane. *Tetrahedron* **1968**, *24*, 1083–1096.

(65) Matczak, P. Theoretical Investigation of the N→Sn Coordination in $(Me_3SnCN)_2$. *Struct. Chem.* **2015**, *26*, 301–318.
